# Supplementary material for: Development modeling of Lucilia sericata (Diptera: Calliphoridae)
Source: PeerJ. 2015 Mar 3;3:e803. doi: 10.7717/peerj.803 (PMC4358662; doi:10.7717/peerj.803)
Supplement: Appendix S1 [file peerj-03-803-s001.pdf]

APPENDIX A

Development data for *Lucilia sericata* (from Excel).

Transitions at 50% by Dose Analysis							
Temp		Hours to Stage Transition					
Mean	SE	E-L1	E-L2	E-L3f	E-L3m	E-P	E-A
10.4	0.51	155.0	317.3	451.5	683.8	1380.2	3798.4
12.7	0.18	32.3	136.1	230.5	431.3	1970.6	2891.0
15.1	0.14	51.7	137.6	179.1	313.8	754.0	1708.5
17.5	0.12	45.6	85.4	136.6	237.2	326.4	760.8
20.1	0.09	28.0	57.9	92.1	189.3	217.0	553.4
22.5	0.10	22.1	45.7	77.5	141.1	202.4	424.6
25.0	0.06	16.9	38.4	62.3	111.3	167.8	370.2
27.5	0.06	11.8	30.9	46.5	124.3	157.6	344.5
30.0	0.05	11.4	24.6	41.6	86.7	139.3	297.2
32.5	0.02	9.7	19.2	35.6	84.5	156.8	308.4

Temp		Hours in Stage					
Mean	SE	Egg	L1	L2	L3f	L3m	P
10.4	0.51	155.0	162.3	134.2	232.3	696.3	2418.3
12.7	0.18	32.3	103.7	94.4	200.8	1539.3	920.4
15.1	0.14	51.7	85.9	41.5	134.7	440.2	954.5
17.5	0.12	45.6	39.8	51.2	100.6	89.2	434.4
20.1	0.09	28.0	29.9	34.2	97.2	27.7	336.4
22.5	0.10	22.1	23.6	31.8	63.6	61.3	222.2
25.0	0.06	16.9	21.5	23.9	49.0	56.4	202.4
27.5	0.06	11.8	19.0	15.6	77.8	33.3	186.9
30.0	0.05	11.4	13.2	17.0	45.1	52.6	157.9
32.5	0.02	9.7	9.5	16.4	48.9	72.3	151.6

Temp		Days to Stage Transition					
Mean	SE	E-L1	E-L2	E-L3f	E-L3m	E-P	E-A
10.4	0.16	6.5	13.2	18.8	28.5	57.5	158.3
12.7	0.13	1.3	5.7	9.6	18.0	82.1	120.5
15.1	0.15	2.2	5.7	7.5	13.1	31.4	71.2
17.5	0.09	1.9	3.6	5.7	9.9	13.6	31.7
20.1	0.1	1.2	2.4	3.8	7.9	9.0	23.1
22.5	0.90	0.9	1.9	3.2	5.9	8.4	17.7
25.0	0.08	0.7	1.6	2.6	4.6	7.0	15.4
27.5	0.07	0.5	1.3	1.9	5.2	6.6	14.4
30.0	0.23	0.5	1.0	1.7	3.6	5.8	12.4
32.5	0.07	0.4	0.8	1.5	3.5	6.5	12.8

Temp		Days in Stage					
Mean	SE	Egg	L1	L2	L3f	L3m	P
10.4	0.16	6.5	6.8	5.6	9.7	29.0	100.8
12.7	0.13	1.3	4.3	3.9	8.4	64.1	38.3
15.1	0.15	2.2	3.6	1.7	5.6	18.3	39.8
17.5	0.09	1.9	1.7	2.1	4.2	3.7	18.1
20.1	0.1	1.2	1.2	1.4	4.0	1.2	14.0
22.5	0.90	0.9	1.0	1.3	2.7	2.6	9.3
25.0	0.08	0.7	0.9	1.0	2.0	2.4	8.4
27.5	0.07	0.5	0.8	0.7	3.2	1.4	7.8
30.0	0.23	0.5	0.5	0.7	1.9	2.2	6.6
32.5	0.07	0.4	0.4	0.7	2.0	3.0	6.3

Temp		1/Days to Stage Transition					
Mean	SE	E-L1	E-L2	E-L3f	E-L3m	E-P	E-A
10.4	0.51	0.155	0.076	0.053	0.035	0.017	0.006
12.7	0.18	0.742	0.176	0.104	0.056	0.012	0.008
15.1	0.14	0.465	0.174	0.134	0.076	0.032	0.014
17.5	0.12	0.526	0.281	0.176	0.101	0.074	0.032
20.1	0.09	0.857	0.414	0.261	0.127	0.111	0.043
22.5	0.10	1.084	0.525	0.310	0.170	0.119	0.057
25.0	0.06	1.418	0.624	0.385	0.216	0.143	0.065
27.5	0.06	2.028	0.778	0.516	0.193	0.152	0.070
30.0	0.05	2.098	0.975	0.577	0.277	0.172	0.081
32.5	0.02	2.470	1.250	0.675	0.284	0.153	0.078

Temp		1/Days in Stage					
Mean	SE	Egg	L1	L2	L3f	L3m	P
10.4	0.51	0.155	0.148	0.179	0.103	0.034	0.010
12.7	0.18	0.742	0.231	0.254	0.120	0.016	0.026
15.1	0.14	0.465	0.279	0.578	0.178	0.055	0.025
17.5	0.12	0.526	0.603	0.469	0.239	0.269	0.055
20.1	0.09	0.857	0.802	0.702	0.247	0.866	0.071
22.5	0.10	1.084	1.017	0.755	0.377	0.392	0.108
25.0	0.06	1.418	1.115	1.006	0.489	0.425	0.119
27.5	0.06	2.028	1.261	1.535	0.308	0.721	0.128
30.0	0.05	2.098	1.823	1.415	0.532	0.456	0.152
32.5	0.02	2.470	2.533	1.465	0.491	0.332	0.158

	Linear Regression Results (from Graph Pad Prism)					
	1/Days to Stage Transition					
	E-L1	E-L2	E-L3f	E-L3m	E-P	E-A
Dev Min (x-intercept):	12.6	10.8	10.5	8.8	10.3	10.7
ADD (1/slope):	8.2	21.3	35.2	82.5	107.5	230.2
Range min:	15.0	15.0	15.0	15.0	17.5	17.5
Range max:	30.0	30.0	30.0	30.0	30.0	30.0

	1/Days in Stage					
	Egg	L1	L2	L3f	L3m	P
Dev Min (x-intercept):	9.5	10.9	9.3	6.6	11.5	10.4
ADD (1/slope):	10.3	11.7	14.3	47.2	29.8	127.9
Range min:	15.0	15.0	15.0	17.5	17.5	17.5
Range max:	30.0	30.0	30.0	30.0	30.0	30.0

Comparison of Transition vs. Stage Results				
	trans	by stage	diff (t-s)	% diff (d/t)
**Egg-L1**	8.2	9.5	-1.3	-15.2%
**L1-L2**	21.3	20.4	1.0	4.5%
**L2-L3f**	35.2	34.6	0.6	1.8%
**L3f-L3m**	82.5	81.8	0.7	0.9%
**L3m-P**	107.5	111.6	-4.1	-3.8%
**P-A**	230.2	239.5	-9.3	-4.0%

From Prism (Linear Regressions): missing values represent non-linearity							
Temp		Transition ADD by 1/Day					
Mean	SE	E-L1	E-L2	E-L3f	E-L3m	E-P	E-A
10.4	0.51						
12.7	0.18	0.14	10.74	21.46	70.59		
15.1	0.14	5.33	24.45	34.36	82.36		
17.5	0.12	9.42	24.00	40.31	86.74	97.81	218.17
20.1	0.09	8.75	22.41	36.95	89.28	88.02	217.35
22.5	0.10	9.18	22.39	39.05	81.06	102.89	210.33
25.0	0.06	8.75	22.75	37.74	75.31	102.40	221.17
27.5	0.06	7.36	21.51	33.07	97.17	112.78	242.08
30.0	0.05	8.31	19.70	33.87	76.76	114.14	239.74
32.5	0.02	8.07	17.36	32.69	83.58		

Temp		Stage ADD by 1/Days					
Mean	SE	Egg	L1	L2	L3f	L3m	P
10.4	0.51						
12.7	0.18	4.3	7.8	13.2	50.8		
15.1	0.14	12.0	14.9	9.9	47.4		
17.5	0.12	15.3	11.0	17.5	45.8	22.3	129.8
20.1	0.09	12.4	11.5	15.3	54.5		136.2
22.5	0.10	12.1	11.5	17.5	42.2	28.1	112.7
25.0	0.06	10.9	12.6	15.6	37.5	31.6	123.4
27.5	0.06	8.9	13.2	11.8	67.7	22.2	133.6
30.0	0.05	9.8	10.5	14.6	44.0	40.5	129.3
32.5	0.02	9.3	8.5	15.8	52.7		

Temp (mean)	Transition ADD by 1/Day					
	E-L1	E-L2	E-L3f	E-L3m	E-P	E-A
10.4						
12.7						
15.1	5.3	24.4	34.4	82.4		
17.5	9.4	24.0	40.3	86.7	97.8	218.2
20.1	8.7	22.4	36.9	89.3	88.0	217.3
22.5	9.2	22.4	39.0	81.1	102.9	210.3
25.0	8.8	22.7	37.7	75.3	102.4	221.2
27.5	7.4	21.5	33.1	97.2	112.8	242.1
30.0	8.3	19.7	33.9	76.8	114.1	239.7
32.5						
**mean**	8.2	22.5	36.5	84.1	103.0	224.8
**SE**	1.3	1.5	2.6	7.1	8.9	11.9
**n**	7	7	7	7	6	6
**Regression ADD**	8	21	35	83	108	230
**% deviation**	-0.8%	5.4%	3.5%	1.9%	-4.2%	-2.3%
**ADD Range min:**	15.0	15.0	15.0	15.0	17.5	17.5
**ADD Range max:**	30.0	30.0	30.0	30.0	30.0	30.0

Temp (mean)	Stage ADD by 1/Days					
	Egg	L1	L2	L3f	L3m	P
10.4						
12.7						
15.1	12.0	14.9	9.9			
17.5	15.3	11.0	17.5	45.8	22.3	129.8
20.1	12.4	11.5	15.3	54.5		136.2
22.5	12.1	11.5	17.5	42.2	28.1	112.7
25.0	10.9	12.6	15.6	37.5	31.6	123.4
27.5	8.9	13.2	11.8	67.7	22.2	133.6
30.0	9.8	10.5	14.6	44.0	40.5	129.3
32.5						
**mean**	11.6	12.2	14.6	48.6	28.9	127.5
**SE**	1.9	1.4	2.6	9.9	6.8	7.7
**n**	7	7	7	6	5	6
**Regression ADD**	10	12	14	47	30	128
**% deviation**	13.3%	4.1%	2.4%	3.1%	-3.0%	-0.3%
**ADD Range min:**	15.0	15.0	15.0	17.5	17.5	17.5
**ADD Range max:**	30.0	30.0	30.0	30.0	30.0	30

Temp	% Time in Stage					
	Egg	L1	L2	L3f	L3m	P
10.4						
12.7	1.1%	3.6%	3.3%	7.0%	53.8%	32.2%
15.1	3.1%	5.2%	2.5%	8.1%	26.6%	57.6%
17.5	6.4%	5.6%	7.2%	14.1%	12.5%	60.7%
20.1	5.3%	5.7%	6.5%	18.5%	5.3%	64.0%
22.5	5.5%	5.9%	7.9%	15.8%	15.2%	55.2%
25.0	4.8%	6.1%	6.8%	13.9%	16.0%	57.3%
27.5	3.6%	5.7%	4.7%	23.4%	10.0%	56.2%
30.0	4.0%	4.6%	5.9%	15.8%	18.4%	55.3%
32.5	3.3%	3.2%	5.5%	16.4%	24.2%	50.8%
**Mean**	4.12%	5.06%	5.58%	14.77%	20.22%	54.37%
**SE**	1.49%	0.98%	1.69%	4.69%	13.42%	8.58%
**CV**	36.14%	19.37%	30.23%	31.76%	66.38%	15.78%

Temp		% Total Development					
Mean	SE	E-L1	E-L2	E-L3f	E-L3m	E-P	E-A
10.4	0.51						
12.7	0.18	0.57%	2.40%	4.07%	7.62%	34.82%	51.08%
15.1	0.14	1.67%	4.45%	5.79%	10.15%	24.38%	55.24%
17.5	0.12	2.95%	5.52%	8.83%	15.34%	21.11%	49.20%
20.1	0.09	2.52%	5.22%	8.30%	17.06%	19.55%	49.87%
22.5	0.10	2.48%	5.13%	8.70%	15.83%	22.71%	47.63%
25.0	0.06	2.26%	5.13%	8.31%	14.85%	22.37%	49.36%
27.5	0.06	1.68%	4.39%	6.61%	17.66%	22.39%	48.95%
30.0	0.05	1.94%	4.18%	7.05%	14.71%	23.63%	50.43%
32.5	0.02	1.61%	3.18%	5.89%	13.98%	25.94%	51.02%
	**Mean**	1.97%	4.40%	7.06%	14.13%	24.10%	50.31%
	**SE**	0.66%	0.97%	1.53%	3.06%	4.16%	2.02%
	**CV**	33.39%	22.13%	21.65%	21.69%	17.28%	4.02%

Temp		ADD Temp * Days to Stage Transition					
Mean	SE	E-L1	E-L2	E-L3f	E-L3m	E-P	E-A
10.4	0.51	66.9					
12.7	0.18	14.0	1.0	1.8	3.3	15.1	22.1
15.1	0.14	22.3	0.8	1.0	1.8	4.3	9.7
17.5	0.12	19.7	0.4	0.7	1.2	1.6	3.8
20.1	0.09	12.1	0.2	0.3	0.7	0.8	2.1
22.5	0.10	9.6	0.2	0.3	0.6	0.8	1.7
25.0	0.06	7.3	0.1	0.2	0.3	0.5	1.0
27.5	0.06	5.1	0.1	0.1	0.3	0.4	0.8
30.0	0.05	4.9	0.1	0.1	0.2	0.3	0.7
32.5	0.02	4.2	0.0	0.0	0.1	0.1	0.2

Temp		ADD Temp * Days in Stage					
Mean	SE	Egg	L1	L2	L3f	L3m	P
10.4	0.51						
12.7	0.18	0.2	0.8	0.7	1.5	11.8	7.0
15.1	0.14	0.3	0.5	0.2	0.8	2.5	5.4
17.5	0.12	0.2	0.2	0.3	0.5	0.4	2.2
20.1	0.09	0.1	0.1	0.1	0.4	0.1	1.3
22.5	0.10	0.1	0.1	0.1	0.3	0.2	0.9
25.0	0.06	0.0	0.1	0.1	0.1	0.2	0.5
27.5	0.06	0.0	0.0	0.0	0.2	0.1	0.4
30.0	0.05	0.0	0.0	0.0	0.1	0.1	0.4
32.5	0.02	0.0	0.0	0.0	0.0	0.0	0.1

For Deming Regressions

Temp		Temp * Days to Stage Transition					
Mean	SE	E-L1	E-L2	E-L3f	E-L3m	E-P	E-A
10.4	0.51	3.3	6.8	9.7	14.6	29.5	81.2
12.7	0.18	0.2	1.0	1.8	3.3	15.1	22.1
15.1	0.14	0.3	0.8	1.0	1.8	4.3	9.7
17.5	0.12	0.2	0.4	0.7	1.2	1.6	3.8
20.1	0.09	0.1	0.2	0.3	0.7	0.8	2.1
22.5	0.10	0.1	0.2	0.3	0.6	0.8	1.7
25.0	0.06	0.0	0.1	0.2	0.3	0.5	1.0
27.5	0.06	0.0	0.1	0.1	0.3	0.4	0.8
30.0	0.05	0.0	0.1	0.1	0.2	0.3	0.7
32.5	0.02	0.0	0.0	0.0	0.1	0.1	0.2

Temp		Temp * Days in Stage					
Mean	SE	Egg	L1	L2	L3f	L3m	P
10.4	0.51	3.3	3.5	2.9	5.0	14.9	51.7
12.7	0.18	0.2	0.8	0.7	1.5	11.8	7.0
15.1	0.14	0.3	0.5	0.2	0.8	2.5	5.4
17.5	0.12	0.2	0.2	0.3	0.5	0.4	2.2
20.1	0.09	0.1	0.1	0.1	0.4	0.1	1.3
22.5	0.10	0.1	0.1	0.1	0.3	0.2	0.9
25.0	0.06	0.0	0.1	0.1	0.1	0.2	0.5
27.5	0.06	0.0	0.0	0.0	0.2	0.1	0.4
30.0	0.05	0.0	0.0	0.0	0.1	0.1	0.4
32.5	0.02	0.0	0.0	0.0	0.0	0.0	0.1

Data for Prism polynomial fit for egg development

Temp	Egg
10.4	0.155
12.7	0.742
15.1	0.465
17.5	0.526
20.1	0.857
22.5	1.084
25.0	1.418
27.5	2.028
30.0	2.098
32.5	1.743

APPENDIX B

Datasets and statistical analysis summary of *Lucilia sericata* nonlinear regressions for percent in stage by temperature. Regression equations are :

Gaussian equation (a standard normal curve):

$$y = a \exp\left[-\frac{1}{2}\left(\frac{x-b}{c}\right)^2\right]$$

Modified Gaussian equation (a form of Gaussian curve with a plateau at 100%):

$$y = a \exp\left[-\frac{1}{2}\left(\frac{|x-b|}{c}\right)^d\right]$$

Cumulative Gaussian equation (a form of the Gaussian curve used for adults, to model a sigmoidal increase to a plateau):

$$y = \frac{a}{2}\left[1 + erf\left(\frac{x-b}{\sqrt{2}c}\right)\right]$$

Reversed cumulative Gaussian equation (a form of the cumulative Gaussian equation used for eggs, to model a sigmoidal decrease from a plateau):

$$y = \frac{a}{2}\left[1 - erf\left(\frac{x-b}{\sqrt{2}c}\right)\right]$$

where x = development time (hours) and y = percent in stage. Where “interrupted” is indicated on a regression, data were insufficient to fit a curve (typically this means we obtained too few points in measuring portions of the transition curve). Where “ambiguous” is indicated on a regression, the program could not reach a final solution by iterative (numerical) methods. The analysis is from GraphPad Prism 6.0 which uses the Marquardt and Levenberg approach for non-linear regression.

Appendix B1. *L. sericata* 10.0° C.

***L. sericata* 10.0° C**

Dataset

Time	egg		L1		L2		L3f		L3m		P		A	
	Mean	SE	Mean	SE	Mean	SE	Mean	SE	Mean	SE	Mean	SE	Mean	SE
0.0	100	0	0	0	0	0	0	0	0	0	0	0	0	0
35.1	100	0	0	0	0	0	0	0	0	0	0	0	0	0
70.2	84.5	26.8	15.5	26.8	0	0	0	0	0	0	0	0	0	0
105.3	66.3	17.5	33.7	17.5	0	0	0	0	0	0	0	0	0	0
140.3	64.7	32	35.3	32	0	0	0	0	0	0	0	0	0	0
175.2	43.1	27.1	56.9	27.1	0	0	0	0	0	0	0	0	0	0
231.2	0	0	76.9	40	23.1	40	0	0	0	0	0	0	0	0
287.2	0	0	50	50	50	50	0	0	0	0	0	0	0	0
343.1	0	0	50	50	50	50	0	0	0	0	0	0	0	0
399.1	0	0	0	0	50	50	50	50	0	0	0	0	0	0
455.0	0	0	0	0	38.1	44.2	61.9	44.2	0	0	0	0	0	0
534.0	0	0	0	0	55	45.6	45	45.6	0	0	0	0	0	0
613.0	0	0	0	0	0	0	100	0	0	0	0	0	0	0
692.1	0	0	0	0	0	0	7.1	7.1	92.9	7.1	0	0	0	0
771.1	0	0	0	0	0	0	0	0	100	0	0	0	0	0
850.1	0	0	0	0	0	0	0	0	100	0	0	0	0	0
989.0	0	0	0	0	0	0	0	0	87.5	0	0	0	0	0
1128.0	0	0	0	0	0	0	0	0	50	50	50	50	0	0
1267.0	0	0	0	0	0	0	0	0	66.7	33.3	33.3	33.3	0	0
1406.0	0	0	0	0	0	0	0	0	87.5	12.5	12.5	12.5	0	0
1545.0	0	0	0	0	0	0	0	0	0	0	100	0	0	0
1880.0	0	0	0	0	0	0	0	0	60	40	20	20	20	20
2215.1	0	0	0	0	0	0	0	0	100	0	0	0	0	0
2885.1	0	0	0	0	0	0	0	0	0	0	100	0	0	0
3220.0	0	0	0	0	0	0	0	0	0	0	50	50	50	50
3747.0	0	0	0	0	0	0	0	0	0	0	88.9	15.7	11.1	15.7
4273.0	0	0	0	0	0	0	0	0	0	0	0	0	100	0
4273.0	0	0	0	0	0	0	0	0	0	0	0	0	100	0

Gaussian	L1	L2	L3f	L3m	P
**Best-fit values**					
Amplitude	72.04	55.44	76.81	96.94	152
Mean	233.8	401.9	542.9	1015	1650
SD	88.9	124.6	107.2	297.2	114.4
**Std. Error**					
Amplitude	3.839	4.469	9.41	18.36	129.7
Mean	5.077	12.42	14.37	61.26	65.94
SD	5.115	11.74	14.25	61.21	48.25
**95% Confidence Intervals**					
Amplitude	64.13 to 79.95	46.24 to 64.65	57.43 to 96.19	59.11 to 134.8	-115.1 to 419.2
Mean	223.4 to 244.3	376.3 to 427.5	513.2 to 572.5	889.3 to 1142	1514 to 1786
SD	78.36 to 99.44	100.4 to 148.8	77.83 to 136.5	171.1 to 423.3	15.03 to 213.8
**Goodness of Fit**					
df	25	25	25	25	25
R square	0.9455	0.8722	0.7742	0.482	0.1177
Absolute SS	716.5	1275	3594	23150	23985
Sy.x	5.353	7.143	11.99	30.43	30.97
**Constraints**					
SD	SD > 0.0	SD > 0.0	SD > 0.0	SD > 0.0	SD > 0.0
Number of points					
Analyzed	28	28	28	28	28

Modified Gaussian	L1	L3f
	Ambiguous	Ambiguous
**Best-fit values**		
a	35.37	~ 100.0
b	~ -551.8	~ 613.0
c	~ 943.8	~ 1.000
d	~ 435.2	~ 1.000
**Std. Error**		
a	5.36	~ 3.391e+011
b	~ 5.623e+021	~ 6.402e+029
c	~ 5.624e+021	~ 3.540e+030
d	~ 2.654e+021	~ 8.101e+029
**95% Confidence Intervals**		
a	24.30 to 46.43	(Very wide)
b	(Very wide)	(Very wide)
c	(Very wide)	(Very wide)
d	(Very wide)	(Very wide)
**Goodness of Fit**		
df	24	24
R square	0.5807	0.4719
Absolute SS	5516	8407
Sy.x	15.16	18.72
**Constraints**		
c	c > 0.0	c > 0.0
d	d > 1.000	d > 1.000
Number of points		
Analyzed	28	28

Cumulative Gaussian	A
**Best-fit values**	
Mean	3757
SD	511.2
**Std. Error**	
Mean	105.4
SD	136.2
**95% Confidence Intervals**	
Mean	3540 to 3974
SD	231.1 to 791.3
**Goodness of Fit**	
df	26
R square	0.8213
Absolute SS	3609
Sy.x	11.78
Number of points	
Analyzed	28

Reversed Cumulative Gaussian	egg
**Best-fit values**	
Mean	151.6
SD	66.18
**Std. Error**	
Mean	3.696
SD	5.504
**95% Confidence Intervals**	
Mean	144.0 to 159.2
SD	54.86 to 77.49
**Goodness of Fit**	
df	26
R square	0.9875
Absolute SS	375
Sy.x	3.798
**Runs test**	
Points above curve	4
Points below curve	10
Number of runs	4
P value (runs test)	0.0679
Deviation from Model	Not Significant
Number of points	
Analyzed	28

Appendix B2. *L. sericata* 12.5° C.

L. sericata 12.5° C														
Dataset														
Time	egg		L1		L2		L3f		L3m		P		A	
	Mean	SE	Mean	SE	Mean	SE	Mean	SE	Mean	SE	Mean	SE	Mean	SE
0.0	100	0	0	0	0	0	0	0	0	0	0	0	0	0
11.0	100	0	0	0	0	0	0	0	0	0	0	0	0	0
46.2	22.1	22.3	77.9	22.3	0	0	0	0	0	0	0	0	0	0
81.1	22.6	10.6	77.4	10.6	0	0	0	0	0	0	0	0	0	0
116.2	16.4	8.2	46.4	35.8	37.2	37.2	0	0	0	0	0	0	0	0
151.1	29.2	29.2	2.1	3.6	68.8	27.9	0	0	0	0	0	0	0	0
207.1	0	0	2.3	3.9	72.7	42.2	25	43.3	0	0	0	0	0	0
263.1	0	0	0	0	12.7	15.9	87.3	15.9	0	0	0	0	0	0
319.1	0	0	0	0	27.5	42.1	72.5	42.1	0	0	0	0	0	0
375.1	0	0	0	0	6.3	10.8	93.8	10.8	0	0	0	0	0	0
431.1	0	0	0	0	0	0	52.1	48	47.9	48	0	0	0	0
510.1	0	0	0	0	0	0	0	0	100	0	0	0	0	0
589.0	0	0	0	0	0	0	0	0	100	0	0	0	0	0
668.1	0	0	0	0	0	0	0	0	100	0	0	0	0	0
747.1	0	0	0	0	0	0	0	0	100	0	0	0	0	0
826.1	0	0	0	0	0	0	0	0	95.9	4.3	4.1	4.3	0	0
965.1	0	0	0	0	0	0	0	0	86	1.7	14	1.7	0	0
1104.0	0	0	0	0	0	0	0	0	85.8	15.3	14.2	15.3	0	0
1243.0	0	0	0	0	0	0	0	0	96.5	3.5	3.5	3.5	0	0
1382.0	0	0	0	0	0	0	0	0	89.7	7.4	10.3	7.4	0	0
1521.0	0	0	0	0	0	0	0	0	71.3	33.7	23.3	24.7	5.4	9.3
1856.1	0	0	0	0	0	0	0	0	64.4	28.9	34.2	26.6	1.4	2.4
2191.1	0	0	0	0	0	0	0	0	14.1	24.4	69.4	20.3	16.5	16.8
2526.0	0	0	0	0	0	0	0	0	10.3	9.1	73	6.6	16.7	10.2
2861.0	0	0	0	0	0	0	0	0	11.7	13.6	48.6	15.7	39.7	24.9
3196.0	0	0	0	0	0	0	0	0	0	0	0	0	100	0
3196.0	0	0	0	0	0	0	0	0	0	0	0	0	100	0
3196.0	0	0	0	0	0	0	0	0	*12.500**	*21.700**	0	0	100	0
3196.0	0	0	0	0	0	0	0	0	*0.000**	*0.000**	0	0	100	0
3196.0	0	0	0	0	0	0	0	0	*5.600**	*7.900**	0	0	100	0
3196.0	0	0	0	0	0	0	0	0	*13.200**	*15.400**	0	0	100	0
	Gaussian						Modified Gaussian				Cumulative Gaussian		Reversed Cumulative Gaussian	
	L1	L2	L3f	L3m	P		L1	L2	L3f	L3m		A		egg
							Ambiguous	Ambiguous	Ambiguous	Ambiguous				
	Best-fit values						Best-fit values				Best-fit values		Best-fit values	
Amplitude	90.67	86.28	95.07	109.6	79.6	a	77.65	~ 72.70	~ 93.80	92.52	Mean	2892	Mean	39.5
Mean	72.97	179.8	333.8	1090	2335	b	~ 66.09	~ 207.1	~ 375.1	~ 1145	SD	124.1	SD	8.719
SD	32.45	45.25	84	475.9	394.3	c	~ 50.07	~ 1.000	~ 1.000	~ 712.6	Std. Error		Std. Error	
	Std. Error					d	~ 42.12	~ 1.000	~ 1.000	~ 164.8	Mean	16.74	Mean	64.9
Amplitude	4.541	5.954	4.958	10.14	5.412		Std. Error				SD	42.29	SD	84.38
Mean	1.879	3.078	5.201	57	30.16	a	0.4265	~ 5.021e+012	~ 1.992e+017	2.446	95% Confidence Intervals		95% Confidence Intervals	
SD	1.78	3.431	5.317	53.18	24.63	b	~ 3.055e+014	~ 4.554e+011	~ 1.951e+014	~ 2.108e+006	Mean	2858 to 2926	Mean	-93.22 to 172.2
	95% Confidence Intervals					c	~ 3.096e+014	~ 2.230e+018	~ 2.551e+022	~ 162266	SD	37.65 to 210.6	SD	-163.8 to 181.3
Amplitude	81.37 to 99.97	74.08 to 98.47	84.92 to 105.2	88.64 to 130.5	68.52 to 90.68	d	~ 5.174e+015	~ 5.541e+017	~ 6.338e+021	~ 2.692e+008	Goodness of Fit		Goodness of Fit	
Mean	69.13 to 76.82	173.5 to 186.1	323.2 to 344.5	972.2 to 1207	2273 to 2397		95% Confidence Intervals				df	29	Degrees of Freedom	29
SD	28.81 to 36.10	38.22 to 52.27	73.11 to 94.88	366.2 to 585.7	343.8 to 444.7	a	76.77 to 78.53	(Very wide)	(Very wide)	87.46 to 97.58	R square	0.9877	R square	0.9159
	Goodness of Fit					b	(Very wide)	(Very wide)	(Very wide)	(Very wide)	Absolute SS	580.2	Absolute SS	1632
df	28	28	28	24	28	c	(Very wide)	(Very wide)	(Very wide)	(Very wide)	Sy.x	4.473	Sy.x	7.503
R square	0.9493	0.9199	0.9438	0.7831	0.8959	d	(Very wide)	(Very wide)	(Very wide)	(Very wide)			Runs test	
Absolute SS	651.2	859.3	1207	10971	1244		Goodness of Fit				Number of points		Points above curve	5
Sy.x	4.822	5.54	6.565	21.38	6.667	df	27	27	27	23	Analyzed	31	Points below curve	1
	Constraints					R square	0.9992	0.3403	0.2453	0.9755			Number of runs	3
SD	SD > 0.0	SD > 0.0	SD > 0.0	SD > 0.0	SD > 0.0	Absolute SS	9.825	7075	16217	1239			P value (runs test)	1
						Sy.x	0.6032	16.19	24.51	7.339			Deviation from Model	Not Significant
Number of points							Constraints							
Analyzed	31	31	31	27	31	c	c > 0.0	c > 0.0	c > 0.0	c > 0.0			Number of points	
						d	d > 1.000	d > 1.000	d > 1.000	d > 1.000			Analyzed	31
						Number of points								
						Analyzed	31	31	31	27				

Appendix B3. *L. sericata* 15.0° C.

***L. sericata* 15.0° C**														
Dataset														
Time	egg		L1		L2		L3f		L3m		P		A	
	Mean	SE	Mean	SE	Mean	SE	Mean	SE	Mean	SE	Mean	SE	Mean	SE
0.0	100	0	0	0	0	0	0	0	0	0	0	0	0	0
17.2	100	0	0	0	0	0	0	0	0	0	0	0	0	0
34.2	100	0	0	0	0	0	0	0	0	0	0	0	0	0
51.2	51.8	19.3	48.2	19.3	0	0	0	0	0	0	0	0	0	0
68.2	6.8	11.8	93.2	11.8	0	0	0	0	0	0	0	0	0	0
97.3	0	0	100	0	0	0	0	0	0	0	0	0	0	0
113.2	0	0	100	0	0	0	0	0	0	0	0	0	0	0
141.1	0	0	4.2	7.2	95.8	7.2	0	0	0	0	0	0	0	0
169.1	0	0	0	0	72.9	42.2	27.1	42.2	0	0	0	0	0	0
197.1	0	0	0	0	16.2	4.6	83.8	4.6	0	0	0	0	0	0
225.1	0	0	0	0	0	0	100	0	0	0	0	0	0	0
264.1	0	0	0	0	0	0	100	0	0	0	0	0	0	0
303.1	0	0	0	0	0	0	93.8	10.8	6.3	10.8	0	0	0	0
318.0	0	0	0	0	0	0	25.9	29.2	74.1	29.2	0	0	0	0
381.1	0	0	0	0	0	0	0	0	100	0	0	0	0	0
444.1	0	0	0	0	0	0	0	0	95	8.7	5	8.7	0	0
491.1	0	0	0	0	0	0	0	0	97	4.3	3	4.3	0	0
564.6	0	0	0	0	0	0	0	0	83.7	12.1	16.3	12.1	0	0
633.0	0	0	0	0	0	0	0	0	61.5	19.9	38.5	19.9	0	0
704.1	0	0	0	0	0	0	0	0	65.7	14.3	34.3	14.3	0	0
775.0	0	0	0	0	0	0	0	0	95	8.7	5	8.7	0	0
942.2	0	0	0	0	0	0	0	0	56.3	39.9	15.1	1.1	28.6	40.4
1108.9	0	0	0	0	0	0	0	0	38.5	24.5	34.9	21.8	26.6	16.6
1277.6	0	0	0	0	0	0	0	0	32.6	20.5	56.2	24.7	11.2	14.9
1443.0	0	0	0	0	0	0	0	0	30.7	26.7	64.1	24.2	5.3	9.1
1610.1	0	0	0	0	0	0	0	0	23.9	23.9	51.5	21.9	24.6	29.5
1873.0	0	0	0	0	0	0	0	0	6.3	10.8	9.4	16.2	84.4	16.2
2135.0	0	0	0	0	0	0	0	0	0	0	0	0	100	0
2398.1	0	0	0	0	0	0	0	0	0	0	0	0	100	0
2664.5	0	0	0	0	0	0	0	0	0	0	0	0	100	0
2664.5	0	0	0	0	0	0	0	0	0	0	0	0	100	0

Gaussian						Modified Gaussian					Cumulative Gaussian		Reversed Cumulative Gaussian	
	L1	L2	L3f	L3m	P		L1	L2	L3f	L3m		A		egg
								Ambiguous						Ambiguous
Best-fit values						Best-fit values					Best-fit values		Best-fit values	
Amplitude	120.7	122.6	116.1	92.69	55.07	a	98.92	~ 95.80	94.63	81.77	Mean	1715	Mean	~ 61.40
Mean	89.52	152.8	249.3	675.2	1348	b	91.24	~ 141.1	243.5	708.8	SD	161.3	SD	~ 0.5224
SD	26.55	16.29	50.55	269.3	390.2	c	37.81	~ 1.001	71.33	398.2	Std. Error		Std. Error	
Std. Error						d	6.512	~ 1.000	21.86	87.09	Mean	29.42	Mean	~
Amplitude	5.753	6.717	6.388	10.25	5.366	Std. Error					SD	34	SD	~
Mean	1.304	0.515	2.785	40.7	47.87	a	0.8761	~ 45925	1.474	4.74	95% Confidence Intervals		95% Confidence Intervals	
SD	1.427	1.097	3.031	33.95	41.7	b	0.6863	~ 2.439e+006	0.185	2.277	Mean	1655 to 1775	Mean	(Very wide)
95% Confidence Intervals						c	0.9975	~ 1.617e+008	2.008	3.096	SD	91.77 to 230.8	SD	(Very wide)
Amplitude	109.0 to 132.5	108.8 to 136.4	103.0 to 129.2	71.70 to 113.7	44.08 to 66.06	d	0.9219		14.22	55.7	Goodness of Fit		Goodness of Fit	
Mean	86.85 to 92.19	151.8 to 153.9	243.6 to 255.0	591.9 to 758.6	1250 to 1446	95% Confidence Intervals					df	29	df	29
SD	23.63 to 29.48	14.04 to 18.53	44.34 to 56.76	199.7 to 338.8	304.8 to 475.6	a	97.12 to 100.7	(Very wide)	91.61 to 97.66	72.05 to 91.50	R square	0.9573	R square	0.9171
Goodness of Fit						b	89.83 to 92.65	(Very wide)	243.1 to 243.9	704.2 to 713.5	Absolute SS	1646	Absolute SS	2369
df	28	28	28	28	28	c	35.76 to 39.86	(Very wide)	67.21 to 75.45	391.8 to 404.5	Sy.x	7.533	Sy.x	9.039
R square	0.9608	0.9841	0.9433	0.6969	0.7889	d	4.620 to 8.404		1.000 to 51.04	1.000 to 201.4			Runs test	
Absolute SS	1065	217.2	1770	12469	2291	Goodness of Fit					Number of points		Points above curve	1
Sy.x	6.168	2.785	7.951	21.1	9.046	df	27	27	27	27	Analyzed	31	Points below curve	1
Constraints						R square	0.9992	0.5915	0.9944	0.882			Number of runs	2
SD	SD > 0.0	SD > 0.0	SD > 0.0	SD > 0.0	SD > 0.0	Absolute SS	22.16	5577	174.9	4852			P value (runs test)	1
						Sy.x	0.906	14.37	2.545	13.41			Deviation from Model	Not Significant
Number of points						Constraints								
Analyzed	31	31	31	31	31	c	c > 0.0	c > 0.0	c > 0.0	c > 0.0			Number of points	
						d	d > 1.000	d > 1.000	d > 1.000	d > 1.000			Analyzed	31
						Number of points								
						Analyzed	31	31	31	31				

Appendix B4. *L. sericata* 17.5° C.

L. sericata 17.5° C

Time	egg Mean	egg SE	L1 Mean	L1 SE	L2 Mean	L2 SE	L3f Mean	L3f SE	L3m Mean	L3m SE	P Mean	P SE	A Mean	A SE
0.0	100	0	0	0	0	0	0	0	0	0	0	0	0	0
12.1	100	0	0	0	0	0	0	0	0	0	0	0	0	0
24.2	100	0	0	0	0	0	0	0	0	0	0	0	0	0
36.2	55.8	21.5	44.2	21.5	0	0	0	0	0	0	0	0	0	0
48.3	54.8	19.4	45.2	19.4	0	0	0	0	0	0	0	0	0	0
60.2	65	28.3	35	28.3	0	0	0	0	0	0	0	0	0	0
79.1	0	0	91.2	15.3	8.8	15.3	0	0	0	0	0	0	0	0
98.2	0	0	1.4	2.4	98.6	2.4	0	0	0	0	0	0	0	0
117.1	0	0	0	0	93.8	10.8	6.3	10.8	0	0	0	0	0	0
136.1	0	0	0	0	53.1	47.1	46.9	47.1	0	0	0	0	0	0
155.1	0	0	0	0	0	0	100	0	0	0	0	0	0	0
181.1	0	0	0	0	0	0	100	0	0	0	0	0	0	0
207.1	0	0	0	0	0	0	93.8	10.8	6.3	10.8	0	0	0	0
233.1	0	0	0	0	0	0	57.1	31.6	42.9	31.6	0	0	0	0
259.1	0	0	0	0	0	0	17.5	20.5	82.5	20.5	0	0	0	0
285.2	0	0	0	0	0	0	0	0	97.2	4.8	2.8	4.8	0	0
333.1	0	0	0	0	0	0	0	0	30.4	34.2	69.6	34.2	0	0
381.1	0	0	0	0	0	0	0	0	26.4	35.7	73.6	35.7	0	0
429.1	0	0	0	0	0	0	0	0	6.3	10.8	93.8	10.8	0	0
477.1	0	0	0	0	0	0	0	0	10.7	11.8	89.3	11.8	0	0
525.1	0	0	0	0	0	0	0	0	29.9	31.8	70.1	31.8	0	0
635.0	0	0	0	0	0	0	0	0	5.8	6.1	94.2	6.1	0	0
745.1	0	0	0	0	0	0	0	0	6.7	9.4	49	38.4	44.3	38
855.0	0	0	0	0	0	0	0	0	16.7	16.7	0	0	83.3	16.7
965.1	0	0	0	0	0	0	0	0	0	0	0	0	100	0
1075.0	0	0	0	0	0	0	0	0	3.6	6.2	0	0	96.4	6.2
1268.2	0	0	0	0	0	0	0	0	0	0	0	0	100	0
1427.0	0	0	0	0	0	0	0	0	0	0	0	0	100	0
1603.0	0	0	0	0	0	0	0	0	0	0	0	0	100	0
1780.8	0	0	0	0	0	0	0	0	0	0	0	0	100	0
1923.5	0	0	0	0	0	0	0	0	0	0	0	0	100	0

Gaussian

	L1	L2	L3	L3m	P
Best-fit values					
Amplitude	69.53	110.8	112.5	98.06	100.7
Mean	68.86	111.1	185.6	280.5	539.1
SD	20.41	18.08	37.58	35.81	151.5
Std. Error					
Amplitude	8.814	4.498	4.179	7.248	7.289
Mean	2.773	0.8477	1.534	3.514	13.25
SD	2.853	0.8529	1.524	3.061	11.74
95% Confidence Intervals					
Amplitude	51.48 to 87.58	101.6 to 120.0	104.0 to 121.1	83.21 to 112.9	85.80 to 115.7
Mean	63.18 to 74.54	109.3 to 112.8	182.4 to 188.7	273.3 to 287.7	512.0 to 566.2
SD	14.57 to 26.26	16.34 to 19.83	34.45 to 40.70	29.54 to 42.08	127.4 to 175.5
Goodness of Fit					
df	28	28	28	28	28
R square	0.7551	0.9671	0.9707	0.8773	0.8875
Absolute SS	2944	636.9	845.5	2069	3795
Sy.x	10.25	4.769	5.495	8.596	11.64
Constraints					
SD	SD > 0.0	SD > 0.0	SD > 0.0	SD > 0.0	SD > 0.0
Number of points					
Analyzed	31	31	31	31	31

Modified Gaussian

	L1	L2	L3	L3m	P
	Hit constraint	Ambiguous			
Best-fit values					
a	119.3	96.2	101.5	102.6	94.2
b	73.47	~ 108.9	186.6	279.7	414.9
c	7.883	~ 26.94	45.85	31.39	9.889
d	~ 1.000	~ 15.67	3.631	1.556	1
Std. Error					
a	87.66	0.6647	2.419	12.06	3149
b	2.543	~ 2892	0.7942	4.164	101.2
c	11	~ 3539	1.185	8.701	463.5
d		~ 35868	0.3222	0.515	19.93
95% Confidence Intervals					
a	-60.58 to 299.2	94.84 to 97.56	96.58 to 106.5	77.81 to 127.3	-6368 to 6556
b	68.26 to 78.69	(Very wide)	184.9 to 188.2	271.2 to 288.3	207.1 to 622.6
c	0.0 to 30.45	(Very wide)	43.42 to 48.28	13.54 to 49.25	0.0 to 961.1
d		(Very wide)	2.970 to 4.292	1.000 to 2.613	1.000 to 41.90
Goodness of Fit					
df	27	27	27	27	27
R square	0.7916	0.9994	0.9902	0.8789	0.005956
Absolute SS	2506	11.52	282.1	2043	33539
Sy.x	9.633	0.6532	3.232	8.698	35.24
Constraints					
c	c > 0.0	c > 0.0	c > 0.0	c > 0.0	c > 0.0
d	d > 1.000	d > 1.000	d > 1.000	d > 1.000	d > 1.000
Number of points					
Analyzed	31	31	31	31	31

Cumulative Gaussian

	A
Best-fit values	
Mean	764.2
SD	82.17
Std. Error	
Mean	2.771
SD	4.003
95% Confidence Intervals	
Mean	758.6 to 769.9
SD	73.99 to 90.36
Goodness of Fit	
df	29
R square	0.9988
Absolute SS	69.74
Sy.x	1.551
Number of points	
Analyzed	31

Reversed Cumulative Gaussian

	egg
Best-fit values	
Mean	54.12
SD	24.41
Std. Error	
Mean	2.495
SD	3.671
95% Confidence Intervals	
Mean	49.02 to 59.22
SD	16.90 to 31.92
Goodness of Fit	
df	29
R square	0.9555
Absolute SS	1470
Sy.x	7.12
Runs test	
Points above curve	4
Points below curve	10
Number of runs	4
P value (runs test)	0.0679
Deviation from Model	Not Significant
Number of points	
Analyzed	31

Appendix B5. *L. sericata* 20.0° C.

L. sericata 20.0° C														
Dataset														
Time	egg		L1		L2		L3f		L3m		P		A	
	Mean	SE	Mean	SE	Mean	SE	Mean	SE	Mean	SE	Mean	SE	Mean	SE
0.0	100	0	0	0	0	0	0	0	0.000*	0.000*	0	0	0	0
9.1	100	0	0	0	0	0	0	0	0.000*	0.000*	0	0	0	0
18.1	100	0	0	0	0	0	0	0	0.000*	0.000*	0	0	0	0
27.3	71.4	17.7	28.6	17.7	0	0	0	0	0.000*	0.000*	0	0	0	0
36.1	77.3	20.7	22.7	20.7	0	0	0	0	0.000*	0.000*	0	0	0	0
45.2	65.8	23.5	34.2	23.5	0	0	0	0	0.000*	0.000*	0	0	0	0
59.1	0	0	12.5	21.7	87.5	21.7	0	0	0.000*	0.000*	0	0	0	0
73.2	0	0	0	0	100	0	0	0	0.000*	0.000*	0	0	0	0
87.1	0	0	0	0	88.7	15.5	11.3	15.5	0.000*	0.000*	0	0	0	0
101.1	0	0	0	0	3.1	5.4	96.9	5.4	0.000*	0.000*	0	0	0	0
115.1	0	0	0	0	0	0	100	0	0.000*	0.000*	0	0	0	0
135.1	0	0	0	0	0	0	100	0	0.000*	0.000*	0	0	0	0
155.1	0	0	0	0	0	0	100	0	0.000*	0.000*	0	0	0	0
175.1	0	0	0	0	0	0	100	0	0	0	0	0	0	0
195.0	0	0	0	0	0	0	9.4	16.2	90.6	16.2	0	0	0	0
215.0	0	0	0	0	0	0	0	0	87.5	21.7	12.5	21.7	0	0
251.0	0	0	0	0	0	0	0	0	0	0	100	0	0	0
287.0	0	0	0	0	0	0	0	0	0	0	100	0	0	0
323.0	0	0	0	0	0	0	0	0	0.000*	0.000*	100	0	0	0
359.0	0	0	0	0	0	0	0	0	0.000*	0.000*	100	0	0	0
395.0	0	0	0	0	0	0	0	0	0.000*	0.000*	100	0	0	0
479.0	0	0	0	0	0	0	0	0	0.000*	0.000*	100	0	0	0
563.0	0	0	0	0	0	0	0	0	0.000*	0.000*	10	17.3	90	17.3
647.0	0	0	0	0	0	0	0	0	0.000*	0.000*	0	0	100	0
731.0	0	0	0	0	0	0	0	0	0.000*	0.000*	3.7	3.7	96.3	3.7
815.0	0	0	0	0	0	0	0	0	0.000*	0.000*	0	0	100	0
815.0	0	0	0	0	0	0	0	0	0.000*	0.000*	0	0	100	0
815.0	0	0	0	0	0	0	0	0	0.000*	0.000*	0	0	100	0
815.0	0	0	0	0	0	0	0	0	0.000*	0.000*	0	0	100	0
815.0	0	0	0	0	0	0	0	0	0.000*	0.000*	0	0	100	0
815.0	0	0	0	0	0	0	0	0	0.000*	0.000*	0	0	100	0

Gaussian					
	L1	L2	L3	L3m	P
				Interrupted	
Best-fit values					
Amplitude	32.59	115.4	120.7	1147	118.2
Mean	41.17	73.37	139.4	205	378.8
SD	13.08	14.37	33.21	4.423	102.6
Std. Error					
Amplitude	2.206	6.131	8.594		8.421
Mean	1.131	0.8848	2.578		9.715
SD	1.069	0.8646	2.58		8.316
95% Confidence Intervals					
Amplitude	28.08 to 37.11	102.8 to 127.9	103.1 to 138.3		101.0 to 135.5
Mean	38.85 to 43.49	71.56 to 75.18	134.1 to 144.7		358.9 to 398.7
SD	10.89 to 15.27	12.60 to 16.14	27.92 to 38.49		85.57 to 119.6
Goodness of Fit					
df	28	28	28		28
R square	0.9065	0.9438	0.899		0.8859
Absolute SS	219.7	1294	4139		5435
Sy.x	2.801	6.799	12.16		13.93
Constraints					
SD	SD > 0.0	SD > 0.0	SD > 0.0	SD > 0.0	SD > 0.0
Number of points					
Analyzed	31	31	31	5	31

Modified Gaussian					
	L1	L2	L3	L3m	P
	Ambiguous			Interrupted	Ambiguous
Best-fit values					
a	28.5	99.94	100.1	9583	100
b	~ 39.28	73.35	140.9	205	~ 388.9
c	~ 19.60	18.42	48.43	1.069	~ 171.4
d	~ 43.39	5.038	14.03	~ 1.000	~ 96.83
Std. Error					
a	1.107	0.3725	0.03356		0.3184
b	~ 6.665e+006	0.06358	0.007575		~ 86811
c	~ 8.810e+006	0.1011	0.03018		~ 2.476e+006
d	~ 4.270e+008	0.07768	0.07961		~ 9.178e+007
95% Confidence Intervals					
a	26.23 to 30.77	99.18 to 100.7	100.0 to 100.2		99.35 to 100.7
b	(Very wide)	73.22 to 73.48	140.9 to 140.9		(Very wide)
c	(Very wide)	18.21 to 18.63	48.37 to 48.50		(Very wide)
d	(Very wide)	4.879 to 5.197	13.87 to 14.20		(Very wide)
Goodness of Fit					
df	27	27	27		27
R square	0.9718	0.9998	1		0.9997
Absolute SS	66.14	3.75	0.1103		13.69
Sy.x	1.565	0.3727	0.06391		0.7121
Constraints					
c	c > 0.0	c > 0.0	c > 0.0	c > 0.0	c > 0.0
d	d > 1.000	d > 1.000	d > 1.000	d > 1.000	d > 1.000
Number of points					
Analyzed	31	31	31	5	31

Cumulative Gaussian	
	A
	Ambiguous
Best-fit values	
Mean	~ 546.2
SD	~ 13.09
Std. Error	
Mean	~ 23306
SD	~ 18184
95% Confidence Intervals	
Mean	(Very wide)
SD	(Very wide)
Goodness of Fit	
df	29
R square	0.9998
Absolute SS	13.69
Sy.x	0.6871
Number of points	
Analyzed	31

Reversed Cumulative Gaussian	
	egg
Best-fit values	
Mean	45.36
SD	13.15
Std. Error	
Mean	1.263
SD	1.78
95% Confidence Intervals	
Mean	42.78 to 47.95
SD	9.504 to 16.79
Goodness of Fit	
df	29
R square	0.9765
Absolute SS	867
Sy.x	5.468
Runs test	
Points above curve	5
Points below curve	7
Number of runs	4
P value (runs test)	0.0758
Deviation from Model	Not Significant
Number of points	
Analyzed	31

Appendix B6. *L. sericata* 22.5 C.

L. sericata **22.5° C**														
Dataset														
Time	egg		L1		L2		L3f		L3m		P		A	
	Mean	SE	Mean	SE	Mean	SE	Mean	SE	Mean	SE	Mean	SE	Mean	SE
0.0	100	0	0	0	0	0	0	0	0	0	0	0	0	0
7.1	100	0	0	0	0	0	0	0	0	0	0	0	0	0
14.2	95	5	5	5	0	0	0	0	0	0	0	0	0	0
21.2	64.6	20.5	35.4	20.5	0	0	0	0	0	0	0	0	0	0
28.2	2.5	4.3	97.5	4.3	0	0	0	0	0	0	0	0	0	0
35.1	8.3	14.4	91.7	14.4	0	0	0	0	0	0	0	0	0	0
46.1	0	0	28.6	30.9	71.4	30.9	0	0	0	0	0	0	0	0
57.1	0	0	0	0	100	0	0	0	0	0	0	0	0	0
68.1	0	0	0	0	100	0	0	0	0	0	0	0	0	0
79.1	0	0	0	0	35.3	24.9	64.7	24.9	0	0	0	0	0	0
90.1	0	0	0	0	5.2	5.3	94.8	5.3	0	0	0	0	0	0
106.1	0	0	0	0	0	0	100	0	0	0	0	0	0	0
122.1	0	0	0	0	0	0	100	0	0	0	0	0	0	0
138.1	0	0	0	0	0	0	40.6	26.8	59.4	26.8	0	0	0	0
154.2	0	0	0	0	0	0	33.3	47.1	66.7	47.1	0	0	0	0
170.1	0	0	0	0	0	0	15	15	85	15	0	0	0	0
198.1	0	0	0	0	0	0	21.8	10.9	41.7	31.2	36.5	27.6	0	0
226.1	0	0	0	0	0	0	0	0	5.6	5.6	94.4	5.6	0	0
254.0	0	0	0	0	0	0	0	0	0	0	100	0	0	0
282.0	0	0	0	0	0	0	0	0	0	0	100	0	0	0
310.0	0	0	0	0	0	0	0	0	0	0	100	0	0	0
377.0	0	0	0	0	0	0	0	0	0	0	100	0	0	0
443.5	0	0	0	0	0	0	0	0	0	0	3.3	3.3	96.7	3.3
511.0	0	0	0	0	0	0	0	0	0	0	2	2.8	98	2.8
578.0	0	0	0	0	0	0	0	0	0	0	6.7	4.1	93.3	4.1
651.0	0	0	0	0	0	0	0	0	0	0	0	0	100	0
651.0	0	0	0	0	0	0	0	0	0	0	0	0	100	0
651.0	0	0	0	0	0	0	0	0	0	0	0	0	100	0
651.0	0	0	0	0	0	0	0	0	0	0	0	0	100	0
651.0	0	0	0	0	0	0	0	0	0	0	0	0	100	0
651.0	0	0	0	0	0	0	0	0	0	0	0	0	100	0

Gaussian					
	L1	L2	L3	L3m	P
Best-fit values					
Amplitude	103.4	113.5	112.1	85.31	118.6
Mean	32.29	60.54	108.7	167.6	308.2
SD	8.003	12.45	23.44	24.89	73.45
Std. Error					
Amplitude	1.858	3.709	6.731	3.8	7.091
Mean	0.1982	0.4702	1.533	1.387	5.569
SD	0.1808	0.4637	1.524	1.287	4.951
95% Confidence Intervals					
Amplitude	99.63 to 107.2	105.9 to 121.1	98.35 to 125.9	77.53 to 93.09	104.1 to 133.2
Mean	31.88 to 32.69	59.58 to 61.50	105.5 to 111.8	164.8 to 170.5	296.8 to 319.6
SD	7.633 to 8.373	11.50 to 13.40	20.32 to 26.57	22.25 to 27.52	63.31 to 83.59
Goodness of Fit					
df	28	28	28	28	28
R square	0.9926	0.9777	0.9243	0.9581	0.9229
Absolute SS	131.9	519	2233	620.4	3144
Sy.x	2.17	4.305	8.931	4.707	10.6
Constraints					
SD	SD > 0.0	SD > 0.0	SD > 0.0	SD > 0.0	SD > 0.0
Number of points					
Analyzed	31	31	31	31	31

Modified Gaussian					
	L1	L2	L3	L3m	P
	Ambiguous	Ambiguous			
Best-fit values					
a	94.6	100	98.83	75.93	100.1
b	~ 33.57	~ 61.49	107.1	166.1	313.5
c	~ 11.85	16.13	28.63	31.12	107.8
d	~ 15.80	~ 8.352	7.323	6.727	10.27
Std. Error					
a	1.245	1.035	5.245	1.925	0.7012
b	~ 59.24	~ 6.135	1.448	0.9176	1.122
c	~ 445.1	2.252	1.289	0.684	1.69
d	~ 12091	~ 46.30	6.663	2.862	1.088
95% Confidence Intervals					
a	92.05 to 97.15	97.89 to 102.1	88.07 to 109.6	71.98 to 79.88	98.64 to 101.5
b	(Very wide)	(Very wide)	104.2 to 110.1	164.2 to 168.0	311.2 to 315.8
c	(Very wide)	11.51 to 20.75	25.99 to 31.28	29.72 to 32.52	104.3 to 111.3
d	(Very wide)	(Very wide)	1.000 to 21.00	1.000 to 12.60	8.033 to 12.50
Goodness of Fit					
df	27	27	27	27	27
R square	0.9977	0.9988	0.9382	0.9866	0.9988
Absolute SS	41.82	27.04	1822	197.9	48.92
Sy.x	1.245	1.001	8.216	2.707	1.346
Constraints					
c	c > 0.0	c > 0.0	c > 0.0	c > 0.0	c > 0.0
d	d > 1.000	d > 1.000	d > 1.000	d > 1.000	d > 1.000
Number of points					
Analyzed	31	31	31	31	31

Cumulative Gaussian	
	A
Best-fit values	
Mean	~ 424.1
SD	~ 10.53
Std. Error	
Mean	~ 2193
SD	~ 1193
95% Confidence Intervals	
Mean	(Very wide)
SD	(Very wide)
Goodness of Fit	
df	29
R square	0.9992
Absolute SS	48.89
Sy.x	1.298
Number of points	
Analyzed	31

Reversed Cumulative Gaussian	
	egg
Best-fit values	
Mean	22.37
SD	3.204
Std. Error	
Mean	0.1757
SD	0.338
95% Confidence Intervals	
Mean	22.01 to 22.73
SD	2.513 to 3.895
Goodness of Fit	
df	29
R square	0.9969
Absolute SS	89.73
Sy.x	1.759
Runs test	
Points above curve	4
Points below curve	3
Number of runs	6
P value (runs test)	0.9714
Deviation from Model	Not Significant
Number of points	
Analyzed	31

Appendix B7. *L. sericata* 25.0° C.

L. sericata 25.0° C														
	Dataset													
Time	egg		L1		L2		L3f		L3m		P		A	
	Mean	SE	Mean	SE	Mean	SE	Mean	SE	Mean	SE	Mean	SE	Mean	SE
0.0	100	0	0	0	0	0	0	0	0	0	0	0	0	0
6.1	100	0	0	0	0	0	0	0	0	0	0	0	0	0
12.1	100	0	0	0	0	0	0	0	0	0	0	0	0	0
18.3	29.3	23.9	70.7	23.9	0	0	0	0	0	0	0	0	0	0
24.2	5.2	3.3	94.8	3.3	0	0	0	0	0	0	0	0	0	0
30.1	6.6	4.4	93.4	4.4	0	0	0	0	0	0	0	0	0	0
39.2	0	0	12.5	21.7	87.5	21.7	0	0	0	0	0	0	0	0
48.3	0	0	0	0	100	0	0	0	0	0	0	0	0	0
57.1	0	0	0	0	84.8	22.5	15.2	22.5	0	0	0	0	0	0
66.1	0	0	0	0	23.8	10.6	76.2	10.6	0	0	0	0	0	0
75.1	0	0	0	0	0	0	100	0	0	0	0	0	0	0
88.1	0	0	0	0	0	0	100	0	0	0	0	0	0	0
101.0	0	0	0	0	0	0	100	0	0	0	0	0	0	0
114.1	0	0	0	0	0	0	18.8	15.3	81.2	15.3	0	0	0	0
127.1	0	0	0	0	0	0	2.8	4.8	97.2	4.8	0	0	0	0
140.3	0	0	0	0	0	0	1.6	2.7	98.4	2.7	0	0	0	0
164.3	0	0	0	0	0	0	0	0	59.9	24.1	40.1	24.1	0	0
188.1	0	0	0	0	0	0	0	0	9.2	6.1	90.8	6.1	0	0
212.1	0	0	0	0	0	0	0	0	5.4	5.9	94.6	5.9	0	0
236.0	0	0	0	0	0	0	0	0	0	0	100	0	0	0
260.0	0	0	0	0	0	0	0	0	3.1	5.4	96.9	5.4	0	0
316.0	0	0	0	0	0	0	0	0	0	0	100	0	0	0
372.0	0	0	0	0	0	0	0	0	0	0	46.6	24.5	53.4	24.5
428.0	0	0	0	0	0	0	0	0	0	0	3.1	3.1	96.9	3.1
484.0	0	0	0	0	0	0	0	0	0	0	6.9	8.8	93.1	8.8
540.0	0	0	0	0	0	0	0	0	0	0	0	0	100	0
540.0	0	0	0	0	0	0	0	0	0	0	0	0	100	0
540.0	0	0	0	0	0	0	0	0	0	0	0	0	100	0
540.0	0	0	0	0	0	0	0	0	0	0	0	0	100	0
540.0	0	0	0	0	0	0	0	0	0	0	0	0	100	0
540.0	0	0	0	0	0	0	0	0	0	0	0	0	100	0

Gaussian	L1	L2	L3	L3m	P
**Best-fit values**					
Amplitude	107	112	118.4	109.1	116.9
Mean	25.86	48.76	86	138.4	269.3
SD	6.825	9.867	17.02	21.81	70.5
**Std. Error**					
Amplitude	3.563	4.864	5.608	5.564	6.253
Mean	0.2986	0.4977	0.8739	1.383	4.487
SD	0.301	0.4869	0.88	1.287	4.066
**95% Confidence Intervals**					
Amplitude	99.70 to 114.3	102.1 to 122.0	107.0 to 129.9	97.75 to 120.5	104.0 to 129.7
Mean	25.24 to 26.47	47.74 to 49.78	84.21 to 87.79	135.5 to 141.2	260.1 to 278.5
SD	6.209 to 7.442	8.869 to 10.86	15.22 to 18.82	19.17 to 24.44	62.17 to 78.83
**Goodness of Fit**					
df	28	28	28	28	28
R square	0.9764	0.9613	0.9547	0.9456	0.9375
Absolute SS	482.8	874.8	1399	1380	2474
Sy.x	4.152	5.59	7.067	7.02	9.401
**Constraints**					
SD	SD > 0.0	SD > 0.0	SD > 0.0	SD > 0.0	SD > 0.0
Number of points					
Analyzed	31	31	31	31	31

Modified Gaussian	L1	L2	L3	L3m	P
	Ambiguous	Ambiguous			
**Best-fit values**					
a	94.15	93.49	101.7	97.81	98.01
b	27.01	49.58	86.15	137.8	269.2
c	9.584	14	22.45	26.61	98.6
d	5.795	6.173	5.401	8.376	9.427
**Std. Error**					
a	0.1379	1.546	0.7683	1.522	0.8625
b	0.1274	0.277	0.1174	2.121	0.3694
c	0.07418	0.3318	0.1886	2.213	0.6682
d	0.4336	0.7666	0.1951	12.26	1.012
**95% Confidence Intervals**					
a	93.87 to 94.44	90.32 to 96.66	100.2 to 103.3	94.69 to 100.9	96.24 to 99.78
b	26.74 to 27.27	49.01 to 50.15	85.91 to 86.39	133.4 to 142.1	268.4 to 269.9
c	9.432 to 9.736	13.32 to 14.68	22.06 to 22.83	22.07 to 31.15	97.23 to 99.97
d	4.905 to 6.685	4.600 to 7.746	5.001 to 5.801	1.000 to 33.53	7.350 to 11.50
**Goodness of Fit**					
df	27	27	27	27	27
R square	1	0.9953	0.999	0.9951	0.9981
Absolute SS	0.9612	106.9	32.11	124.1	76.43
Sy.x	0.1887	1.99	1.091	2.144	1.682
**Constraints**					
c	c > 0.0	c > 0.0	c > 0.0	c > 0.0	c > 0.0
d	d > 1.000	d > 1.000	d > 1.000	d > 1.000	d > 1.000
Number of points					
Analyzed	31	31	31	31	31

Cumulative Gaussian	A
**Best-fit values**	
Mean	370.1
SD	24.86
Std. Error	
Mean	0.8794
SD	3.293
**95% Confidence Intervals**	
Mean	368.3 to 371.9
SD	18.13 to 31.60
**Goodness of Fit**	
df	29
R square	0.9991
Absolute SS	54.35
Sy.x	1.369
Number of points	
Analyzed	31

Reversed Cumulative Gaussian	egg
**Best-fit values**	
Mean	17.3
SD	1.84
Std. Error	
Mean	0.6314
SD	1.153
**95% Confidence Intervals**	
Mean	16.01 to 18.59
SD	-0.5183 to 4.19
**Goodness of Fit**	
Degrees of Freedom	29
R square	0.9974
Absolute SS	70.56
Sy.x	1.56
**Runs test**	
Points above curve	4
Points below curve	1
Number of runs	3
P value (runs test)	1
Deviation from Model	Not Significant
Number of points	
Analyzed	31

Appendix B8. *L. sericata* 27.5° C.

L. sericata 27.5° C														
Dataset														
Time	egg		L1		L2		L3f		L3m		P		A	
	Mean	SE	Mean	SE	Mean	SE	Mean	SE	Mean	SE	Mean	SE	Mean	SE
0	100	0	0	0	0	0	0	0	0	0	0	0	0	0
5.1	100	0	0	0	0	0	0	0	0	0	0	0	0	0
10.1	51.1	30.2	48.9	30.2	0	0	0	0	0	0	0	0	0	0
15.3	29.9	36	70.1	36	0	0	0	0	0	0	0	0	0	0
20.3	33.1	25.5	66.9	25.5	0	0	0	0	0	0	0	0	0	0
25.2	14.6	9.6	85.4	9.6	0	0	0	0	0	0	0	0	0	0
33.2	0	0	10.7	5.5	89.3	5.5	0	0	0	0	0	0	0	0
41.2	0	0	3.9	4.2	96.1	4.2	0	0	0	0	0	0	0	0
49.2	0	0	0	0	1.5	2.5	98.5	2.5	0	0	0	0	0	0
57.1	0	0	0	0	8.3	10.2	91.7	10.2	0	0	0	0	0	0
65.1	0	0	0	0	1.6	2.7	98.4	2.7	0	0	0	0	0	0
76.1	0	0	0	0	2.8	4.8	97.2	4.8	0	0	0	0	0	0
87.1	0	0	0	0	0	0	100	0	0	0	0	0	0	0
98.1	0	0	0	0	0	0	100	0	0	0	0	0	0	0
110.1	0	0	0	0	0	0	100	0	0	0	0	0	0	0
121	0	0	0	0	0	0	62.5	41.5	37.5	41.5	0	0	0	0
141.1	0	0	0	0	0	0	6.3	10.8	90.6	10.4	3.1	5.4	0	0
161.1	0	0	0	0	0	0	0	0	35	23.7	65	23.7	0	0
181	0	0	0	0	0	0	4.2	7.2	8.3	14.4	87.5	21.7	0	0
201	0	0	0	0	0	0	0	0	0	0	100	0	0	0
221	0	0	0	0	0	0	0	0	0	0	100	0	0	0
269.1	0	0	0	0	0	0	0	0	0	0	100	0	0	0
317	0	0	0	0	0	0	0	0	0	0	75	30.6	25	30.6
365	0	0	0	0	0	0	0	0	16.7	23.6	16.7	23.6	66.7	47.1
413	0	0	0	0	0	0	0	0	0	0	0	0	100	0
461	0	0	0	0	0	0	0	0	0	0	0	0	100	0
461	0	0	0	0	0	0	0	0	0	0	0	0	100	0
461	0	0	0	0	0	0	0	0	0	0	0	0	100	0
461	0	0	0	0	0	0	0	0	0	0	0	0	100	0
461	0	0	0	0	0	0	0	0	0	0	0	0	100	0
461	0	0	0	0	0	0	0	0	0	0	0	0	100	0

Gaussian					
	L1	L2	L3	L3m	P
Best-fit values					
Amplitude	83.21	167.4	117	91.35	120.3
Mean	20.28	37.32	85.61	141.3	245.5
SD	7.864	3.679	28.77	14.33	61.29
Std. Error					
Amplitude	4.436	30.27	8.105	3.789	6.507
Mean	0.5489	0.05786	2.326	0.7781	3.581
SD	0.5365	0.5615	2.338	0.5912	3.446
95% Confidence Intervals					
Amplitude	74.13 to 92.29	105.4 to 229.4	100.4 to 133.6	83.58 to 99.11	106.9 to 133.6
Mean	19.16 to 21.41	37.21 to 37.44	80.84 to 90.37	139.7 to 142.9	238.2 to 252.8
SD	6.765 to 8.962	2.529 to 4.829	23.98 to 33.56	13.12 to 15.54	54.24 to 68.35
Goodness of Fit					
df	28	28	28	28	28
R square	0.9384	0.995	0.8913	0.9592	0.9411
Absolute SS	1021	80.17	5719	409.5	2248
Sy.x	6.039	1.692	14.29	3.824	8.96
Constraints					
SD	SD > 0.0	SD > 0.0	SD > 0.0	SD > 0.0	SD > 0.0
Number of points					
Analyzed	31	31	31	31	31

Modified Gaussian					
	L1	L2	L3	L3m	P
	Ambiguous	Ambiguous			
Best-fit values					
a	74.16	~ 962.6	97.97	90.6	98.24
b	20.7	37.26	~ 81.52	~ 140.9	241.4
c	10.81	~ 0.8543	~ 39.51	~ 18.37	81.36
d	~ 9.333	~ 1.000	~ 177.8	~ 6.915	8.464
Std. Error					
a	2.385	~ 60920	1.222	3.589	1.921
b	11.66	0.6686	~ 3.324e+014	~ 5216	1.071
c	9.344	~ 44.73	~ 3.137e+014	~ 76857	1.103
d	~ 115.6		~ 1.420e+017	~ 330532	1.113
95% Confidence Intervals					
a	69.27 to 79.05	(Very wide)	95.46 to 100.5	83.24 to 97.96	94.30 to 102.2
b	-3.218 to 44.62	35.89 to 38.63	(Very wide)	(Very wide)	239.2 to 243.6
c	0.0 to 29.98	(Very wide)	(Very wide)	(Very wide)	79.10 to 83.62
d	(Very wide)		(Very wide)	(Very wide)	6.181 to 10.75
Goodness of Fit					
df	27	27	27	27	27
R square	0.9873	0.995	0.9979	0.9654	0.9909
Absolute SS	210.5	80.2	110.1	347.8	345.1
Sy.x	2.792	1.724	2.019	3.589	3.575
Constraints					
c	c > 0.0	c > 0.0	c > 0.0	c > 0.0	c > 0.0
d	d > 1.000	d > 1.000	d > 1.000	d > 1.000	d > 1.000
Number of points					
Analyzed	31	31	31	31	31

Cumulative Gaussian	
	A
Best-fit values	
Mean	345.7
SD	38.65
Std. Error	
Mean	0.8633
SD	1.195
95% Confidence Intervals	
Mean	343.9 to 347.4
SD	36.21 to 41.10
Goodness of Fit	
df	29
R square	0.9994
Absolute SS	32.79
Sy.x	1.063
Number of points	
Analyzed	31

Reversed Cumulative Gaussian	
	egg
Best-fit values	
Mean	13.34
SD	8.528
Std. Error	
Mean	0.6601
SD	0.9593
95% Confidence Intervals	
Mean	11.99 to 14.69
SD	6.567 to 10.49
Goodness of Fit	
df	29
R square	0.9617
Absolute SS	817.4
Sy.x	5.309
Runs test	
Points above curve	4
Points below curve	8
Number of runs	4
P value (runs test)	0.1091
Deviation from Model	Not Significant
Number of points	
Analyzed	31

Appendix B9. *L. sericata* 30.0° C.

L. sericata 30.0° C														
	Dataset													
Time	egg		L1		L2		L3f		L3m		P		A	
	Mean	SE	Mean	SE	Mean	SE	Mean	SE	Mean	SE	Mean	SE	Mean	SE
0.0	100	0	0	0	0	0	0	0	0	0	0	0	0	0
4.1	100	0	0	0	0	0	0	0	0	0	0	0	0	0
8.1	86.5	15.6	13.5	15.6	0	0	0	0	0	0	0	0	0	0
12.1	29.7	20.3	70.3	20.3	0	0	0	0	0	0	0	0	0	0
15.3	37.1	15.5	62.9	15.5	0	0	0	0	0	0	0	0	0	0
20.2	54.4	13.8	45.6	13.8	0	0	0	0	0	0	0	0	0	0
27.2	0	0	0	0	100	0	0	0	0	0	0	0	0	0
34.2	0	0	0	0	100	0	0	0	0	0	0	0	0	0
41.3	0	0	0	0	49.6	43.6	50.4	43.6	0	0	0	0	0	0
48.2	0	0	0	0	15	22.3	85	22.3	0	0	0	0	0	0
55.2	0	0	0	0	0	0	100	0	0	0	0	0	0	0
65.1	0	0	0	0	0	0	100	0	0	0	0	0	0	0
75.2	0	0	0	0	0	0	97.4	3.6	2.6	3.6	0	0	0	0
85.1	0	0	0	0	0	0	77.3	39.4	22.7	39.4	0	0	0	0
95.1	0	0	0	0	0	0	0	0	100	0	0	0	0	0
101.1	0	0	0	0	0	0	3.3	5.8	96.7	5.8	0	0	0	0
123.0	0	0	0	0	0	0	0	0	86.5	15.4	13.5	15.4	0	0
141.1	0	0	0	0	0	0	0	0	46.7	31.3	53.3	31.3	0	0
159.3	0	0	0	0	0	0	1.7	2.9	4.5	7.9	93.8	7.4	0	0
177.0	0	0	0	0	0	0	0	0	3.2	3.2	96.8	3.2	0	0
195.0	0	0	0	0	0	0	0	0	1.7	2.9	98.3	2.9	0	0
237.0	0	0	0	0	0	0	0	0	0	0	100	0	0	0
279.0	0	0	0	0	0	0	0	0	0	0	88.7	12.1	11.3	12.1
322.0	0	0	0	0	0	0	0	0	0	0	6.3	10.8	93.8	10.8
363.0	0	0	0	0	0	0	0	0	0	0	18.4	7.8	81.6	7.8
405.0	0	0	0	0	0	0	0	0	0	0	0	0	100	0
405.0	0	0	0	0	0	0	0	0	0	0	0	0	100	0
405.0	0	0	0	0	0	0	0	0	0	0	0	0	100	0
405.0	0	0	0	0	0	0	0	0	0	0	0	0	100	0
405.0	0	0	0	0	0	0	0	0	0	0	0	0	100	0
405.0	0	0	0	0	0	0	0	0	0	0	0	0	100	0

Gaussian					
	L1	L2	L3	L3m	P
Best-fit values					
Amplitude	72.83	115.8	116.7	119	119.9
Mean	15.25	32.23	64.77	111.8	218.8
SD	4.603	6.444	16.43	17.93	56.47
Std. Error					
Amplitude	3.116	5.373	6.105	7.008	6.776
Mean	0.2472	0.3465	0.9292	0.9427	3.453
SD	0.2527	0.3402	0.9256	1.093	3.32
95% Confidence Intervals					
Amplitude	66.45 to 79.21	104.8 to 126.8	104.2 to 129.2	104.6 to 133.3	106.0 to 133.8
Mean	14.75 to 15.76	31.52 to 32.94	62.87 to 66.67	109.9 to 113.7	211.7 to 225.8
SD	4.085 to 5.120	5.748 to 7.141	14.53 to 18.32	15.70 to 20.17	49.67 to 63.27
Goodness of Fit					
df	28	28	28	28	28
R square	0.9627	0.9566	0.9423	0.9465	0.9346
Absolute SS	371.9	885.5	2115	1352	2530
Sy.x	3.645	5.624	8.691	6.949	9.505
Constraints					
SD	SD > 0.0	SD > 0.0	SD > 0.0	SD > 0.0	SD > 0.0
Number of points					
Analyzed	31	31	31	31	31

Modified Gaussian				
	L1	L2	L3	L3m
	Ambiguous	Ambiguous		
Best-fit values				
a	66.6	100	97.92	94.4
b	~ 14.42	~ 31.45	64.92	~ 113.4
c	~ 5.882	~ 9.729	22.43	~ 27.40
d	~ 16.22	~ 26.58	6.59	~ 31.94
Std. Error				
a	1.007	2.887	0.9499	1.606
b	~ 2388	~ 8.079e+006	0.1622	~ 501.7
c	~ 1531	~ 9.121e+006	0.1795	~ 972.1
d	~ 144794	~ 2.829e+008	0.3966	~ 51806
95% Confidence Intervals				
a	64.53 to 68.67	94.08 to 105.9	95.97 to 99.87	91.11 to 97.69
b	(Very wide)	(Very wide)	64.59 to 65.25	(Very wide)
c	(Very wide)	(Very wide)	22.07 to 22.80	(Very wide)
d	(Very wide)	(Very wide)	5.776 to 7.404	(Very wide)
Goodness of Fit				
df	27	27	27	27
R square	0.9973	0.989	0.9979	0.9945
Absolute SS	27.38	225	76.8	139.2
Sy.x	1.007	2.887	1.687	2.271
Constraints				
c	c > 0.0	c > 0.0	c > 0.0	c > 0.0
d	d > 1.000	d > 1.000	d > 1.000	d > 1.000
Number of points				
Analyzed	31	31	31	31

Cumulative Gaussian	
	A
Best-fit values	
Mean	297.9
SD	15.65
Std. Error	
Mean	2.477
SD	1.886
95% Confidence Intervals	
Mean	292.9 to 303.0
SD	11.80 to 19.51
Goodness of Fit	
df	29
R square	0.9939
Absolute SS	338.5
Sy.x	3.416
Number of points	
Analyzed	31

Reversed Cumulative Gaussian	
	egg
Best-fit values	
Mean	14.33
SD	9.125
Std. Error	
Mean	1.038
SD	1.629
95% Confidence Intervals	
Mean	12.20 to 16.45
SD	5.793 to 12.46
Goodness of Fit	
df	29
R square	0.9204
Absolute SS	2176
Sy.x	8.662
Runs test	
Points above curve	4
Points below curve	10
Number of runs	4
P value (runs test)	0.0679
Deviation from Model	Not Significant
Number of points	
Analyzed	31

Appendix B10. *L. sericata* 32.5° C.

L. sericata 32.5° C														
Dataset														
Time	egg		L1		L2		L3f		L3m		P		A	
	Mean	SE	Mean	SE	Mean	SE	Mean	SE	Mean	SE	Mean	SE	Mean	SE
0	100	0	0	0	0	0	0	0	0	0	0	0	0	0
5.1	100	0	0	0	0	0	0	0	0	0	0	0	0	0
10.1	51.1	30.2	48.9	30.2	0	0	0	0	0	0	0	0	0	0
15.3	29.9	36	70.1	36	0	0	0	0	0	0	0	0	0	0
20.3	33.1	25.5	66.9	25.5	0	0	0	0	0	0	0	0	0	0
25.2	14.6	9.6	85.4	9.6	0	0	0	0	0	0	0	0	0	0
33.2	0	0	10.7	5.5	89.3	5.5	0	0	0	0	0	0	0	0
41.2	0	0	3.9	4.2	96.1	4.2	0	0	0	0	0	0	0	0
49.2	0	0	0	0	1.5	2.5	98.5	2.5	0	0	0	0	0	0
57.1	0	0	0	0	8.3	10.2	91.7	10.2	0	0	0	0	0	0
65.1	0	0	0	0	1.6	2.7	98.4	2.7	0	0	0	0	0	0
76.1	0	0	0	0	2.8	4.8	97.2	4.8	0	0	0	0	0	0
87.1	0	0	0	0	0	0	100	0	0	0	0	0	0	0
98.1	0	0	0	0	0	0	100	0	0	0	0	0	0	0
110.1	0	0	0	0	0	0	100	0	0	0	0	0	0	0
121	0	0	0	0	0	0	62.5	41.5	37.5	41.5	0	0	0	0
141.1	0	0	0	0	0	0	6.3	10.8	90.6	10.4	3.1	5.4	0	0
161.1	0	0	0	0	0	0	0	0	35	23.7	65	23.7	0	0
181	0	0	0	0	0	0	4.2	7.2	8.3	14.4	87.5	21.7	0	0
201	0	0	0	0	0	0	0	0	0	0	100	0	0	0
221	0	0	0	0	0	0	0	0	0	0	100	0	0	0
269.1	0	0	0	0	0	0	0	0	0	0	100	0	0	0
317	0	0	0	0	0	0	0	0	0	0	75	30.6	25	30.6
365	0	0	0	0	0	0	0	0	16.7	23.6	16.7	23.6	66.7	47.1
413	0	0	0	0	0	0	0	0	0	0	0	0	100	0
461	0	0	0	0	0	0	0	0	0	0	0	0	100	0
461	0	0	0	0	0	0	0	0	0	0	0	0	100	0
461	0	0	0	0	0	0	0	0	0	0	0	0	100	0
461	0	0	0	0	0	0	0	0	0	0	0	0	100	0
461	0	0	0	0	0	0	0	0	0	0	0	0	100	0
461	0	0	0	0	0	0	0	0	0	0	0	0	100	0

Gaussian					
	L1	L2	L3	L3m	P
Best-fit values					
Amplitude	80.22	107.7	109.6	102.3	90.26
Mean	18.74	29.64	65.21	131.6	263.9
SD	5.087	4.25	21.25	28.73	70.33
Std. Error					
Amplitude	1.648	5.769	7.562	7.385	5.277
Mean	0.122	0.325	1.657	2.233	5.088
SD	0.1204	0.3562	1.684	2.263	4.604
95% Confidence Intervals					
Amplitude	76.84 to 83.59	95.85 to 119.5	94.10 to 125.1	87.14 to 117.4	79.46 to 101.1
Mean	18.49 to 18.99	28.98 to 30.31	61.82 to 68.61	127.0 to 136.2	253.5 to 274.3
SD	4.840 to 5.334	3.521 to 4.979	17.80 to 24.70	24.10 to 33.37	60.90 to 79.76
Goodness of Fit					
df	28	28	28	28	28
R square	0.9914	0.9526	0.8938	0.8888	0.9202
Absolute SS	99.29	630.7	4598	3059	2063
Sy.x	1.883	4.746	12.82	10.45	8.583
Constraints					
SD	SD > 0.0	SD > 0.0	SD > 0.0	SD > 0.0	SD > 0.0
Number of points					
Analyzed	31	31	31	31	31

Modified Gaussian					
	L1	L2	L3	L3m	P
	Ambiguous	Ambiguous			
Best-fit values					
a	78.98	221.8	85.64	96.9	74.75
b	18.75	28.89	~ 65.08	131.6	265.7
c	5.271	1.457	~ 31.60	31.66	101.1
d	2.159	~ 1.000	~ 57.73	2.517	7.427
Std. Error					
a	1.87	657.4	3.952	9.432	3.322
b	0.1218	0.8872	~ 2.390e+007	2.236	5.671
c	0.1889	6.018	~ 2.873e+007	4.289	5.601
d	0.1582		~ 5.303e+008	0.7728	1.875
95% Confidence Intervals					
a	75.14 to 82.82	-1127 to 1571	77.53 to 93.75	77.54 to 116.2	67.93 to 81.57
b	18.50 to 19.00	27.07 to 30.71	(Very wide)	127.0 to 136.2	254.1 to 277.3
c	4.883 to 5.659	0.0 to 13.81	(Very wide)	22.86 to 40.46	89.65 to 112.6
d	1.834 to 2.484		(Very wide)	1.000 to 4.103	3.580 to 11.27
Goodness of Fit					
df	27	27	27	27	27
R square	0.9917	0.9512	0.9513	0.8905	0.955
Absolute SS	95.44	650	2108	3012	1164
Sy.x	1.88	4.907	8.836	10.56	6.567
Constraints					
c	c > 0.0	c > 0.0	c > 0.0	c > 0.0	c > 0.0
d	d > 1.000	d > 1.000	d > 1.000	d > 1.000	d > 1.000
Number of points					
Analyzed	31	31	31	31	31

Cumulative Gaussian	
	A
Best-fit values	
Mean	350.2
SD	49.48
Std. Error	
Mean	5.126
SD	7.146
95% Confidence Intervals	
Mean	339.7 to 360.6
SD	34.86 to 64.09
Goodness of Fit	
df	29
R square	0.978
Absolute SS	905.4
Sy.x	5.587
Number of points	
Analyzed	31

Reversed Cumulative Gaussian	
	egg
Best-fit values	
Mean	14.1
SD	5.046
Std. Error	
Mean	0.1485
SD	0.2359
95% Confidence Intervals	
Mean	13.80 to 14.40
SD	4.563 to 5.528
Goodness of Fit	
df	29
R square	0.9966
Absolute SS	98.4
Sy.x	1.842
Runs test	
Points above curve	5
Points below curve	6
Number of runs	4
P value (runs test)	0.1104
Deviation from Model	Not Significant
Number of points	
Analyzed	31

APPENDIX C

Datasets and statistical analysis summary of *Lucilia sericata* nonlinear regressions for 50% stage transition by temperature. Regression equation:

$$y = 100/(1+10^{((LogEC50-x)*HillSlope)})$$

where x = time (log time) and y = percent in stage. Where “interrupted” is indicated on a regression, data were insufficient to fit a curve (typically this means we obtained too few points in measuring portions of the transition curve). Where “ambiguous” is indicated on a regression, the program could not reach a final solution by iterative (numerical) methods.. The analysis is from GraphPad Prism 6.0 which uses the Marquardt and Levenberg approach for non-linear regression.

Appendix C1. *L. sericata* 10° C.

***L. sericata* 10.0° C**	L1	L2	L3f	L3m	P	A
log(agonist) vs. normalized response -- Variable slope				Interrupted		
Best-fit values						
LogEC50	2.19	2.501	2.655	2.835	3.14	3.58
HillSlope	2.414	4.076	5.869	214.8	6.86	9.414
EC50	155	317.3	451.5	683.8	1380	3798
Std. Error						
LogEC50	0.02321	0.01958	0.02237		0.02109	0.01581
HillSlope	0.3905	0.8776	1.748		2.587	3.207
95% Confidence Intervals						
LogEC50	2.126 to 2.255	2.454 to 2.549	2.605 to 2.705		3.096 to 3.184	3.547 to 3.612
HillSlope	1.330 to 3.498	1.928 to 6.223	1.976 to 9.763		1.424 to 12.30	2.796 to 16.03
EC50	133.7 to 179.8	284.2 to 354.3	402.6 to 506.4		1246 to 1528	3524 to 4095
Goodness of Fit						
Degrees of Freedom	4	6	10		18	24
R square	0.9634	0.9327	0.8665		0.6914	0.7114
Absolute Sum of Squares	140.3	244.8	1717		3656	3395
Sy.x	5.922	6.387	13.1		14.25	11.89
Runs test						
Points above curve	3	2	3		2	3
Points below curve	3	6	9		18	23
Number of runs	4	3	4		4	6
P value (runs test)	0.7	0.2857	0.2		0.2842	0.4077
Deviation from Model	Not Significant	Not Significant	Not Significant		Not Significant	Not Significant
Number of points analyzed	6	8	12	15	20	26
	LogEC50		HillSlope		EC50	
	Value	SE	Value	SE	Value	SE
L1	2.190	0.023	2.414	0.391	155.047	
L2	2.501	0.020	4.076	0.878	317.305	
L3f	2.655	0.022	5.869	1.748	451.532	
L3m	2.835		214.846		683.849	
P	3.140	0.021	6.860	2.587	1380.160	
A	3.580	0.016	9.414	3.207	3798.426	

**Dataset**						
* starred and italicized data points were excluded from final regression						
log(time)	L1	L2	L3f	L3m	P	A
1.545359	0	0	0	0	0	0
1.846131	15.5	0	0	0	0	0
2.022291	33.7	0	0	0	0	0
2.146916	35.3	0	0	0	0	0
2.243462	56.9	0	0	0	0	0
2.36398	76.9	23.1	0	0	0	0
2.458172		50	0	0	0	0
2.535384		50	0	0	0	0
2.601109			50	0	0	0
2.658011			61.9	0	0	0
2.727541			45	0	0	0
2.787475			100	0	0	0
2.840158				92.9	0	0
2.887106				100	0	0
2.929462				100	0	0
2.995196					0	0
3.052309					50	0
3.102777					33.3	0
3.147985					12.5	0
3.188928					100	0
3.274166						20
3.34539						0
3.460155						0
3.507861						50
3.573689						11.1
3.630733						100

Appendix C2. *L. sericata* 12.5° C.

***L. sericata* 12.5° C**	L1	L2	L3f	L3m	P	A
log(agonist) vs. normalized response -- Variable slope				Ambiguous		
Best-fit values						
LogEC50	1.51	2.134	2.363	~ 2.635	3.295	3.461
HillSlope	2.42	4.088	8.158	~ 182.5	6.14	35.54
EC50	32.35	136.1	230.5	~ 431.3	1971	2891
Std. Error						
LogEC50	0.1559	0.02671	0.01772	~ 2.490e-006	0.004932	0.002677
HillSlope	1.574	1.065	2.296	~ 2.273	0.4259	11.44
95% Confidence Intervals						
LogEC50	-0.4711 to 3.491	2.060 to 2.208	2.321 to 2.405	(Very wide)	3.284 to 3.305	3.456 to 3.467
HillSlope	-17.58 to 22.42	1.132 to 7.045	2.727 to 13.59	(Very wide)	5.241 to 7.039	11.92 to 59.16
EC50	0.3380 to 3096	114.7 to 161.4	209.3 to 253.9	(Very wide)	1924 to 2018	2854 to 2928
Goodness of Fit						
Degrees of Freedom	1	4	7	10	17	24
R square	0.933	0.9443	0.9527	1	0.9815	0.9701
Absolute Sum of Squares	269.5	338.8	646.4	7.423E-19	103.5	571.9
Sy.x	16.42	9.203	9.609	2.725E-10	2.467	4.881
Runs test						
Points above curve	1	2	1	2	3	6
Points below curve	2	4	8	9	16	20
Number of runs	3	3	3	2	4	4
P value (runs test)	1	0.4	1	0.0364	0.0815	0.0009
Deviation from Model	Not Significant	Not Significant	Not Significant	Significant	Not Significant	Significant
Number of points						
Analyzed	3	6	9	12	19	26

	LogEC50		HillSlope		EC50	
	Value	SE	Value	SE	Value	SE
L1	1.509841	0.1559078	2.419713	1.573807	32.34751	
L2	2.13381	0.02671435	4.088431	1.064884	136.0847	
L3f	2.362672	0.01772014	8.158384	2.296432	230.5008	
L3m	2.634765	2.49036E-06	182.5313	2.273092	431.2857	
P	3.294597	0.004932407	6.140238	0.4259485	1970.594	
A	3.461043	0.002676541	35.54379	11.444	2890.963	

Dataset						
* starred and italicized data points were excluded from final regression						
log(time)	L1	L2	L3f	L3m	P	A
1.042871	0	0	0	0	0	0
1.664916	77.9	0	0	0	0	0
1.908999	77.4	0	0	0	0	0
2.065113		37.2	0	0	0	0
2.179384		68.8	0	0	0	0
2.316259		72.7	25	0	0	0
2.42019			87.3	0	0	0
2.503932			72.5	0	0	0
2.574147			93.8	0	0	0
2.634565				47.9	0	0
2.707645				100	0	0
2.770115				100	0	0
2.824825					0	0
2.873381					0	0
2.917006					*4.1**	0
2.984578					*14.0**	0
3.042969					*14.2**	0
3.094471					3.5	0
3.140508					10.3	0
3.182129					23.3	5.4
3.26861					34.2	1.4
3.340662					69.4	16.5
3.402433					*73.0**	16.7
3.456518						39.7
3.504607						100
3.504607						100

Appendix C3. *L. sericata* 15.0° C.

***L. sericata* 15.0° C**	L1	L2	L3f	L3m	P	A
log(agonist) vs. normalized response -- Variable slope		Interrupted				
Best-fit values						
LogEC50	1.713	2.138	2.253	2.497	2.877	3.233
HillSlope	9.842	122.8	17.59	78.1	5.488	17.9
EC50	51.66	137.6	179.1	313.8	754	1709
Std. Error						
LogEC50	0.001607		0.0008488	0.0004665	0.01293	0.008174
HillSlope	0.5343		0.4598	5.118	0.7987	4.254
95% Confidence Intervals						
LogEC50	1.709 to 1.718		2.251 to 2.255	2.496 to 2.498	2.850 to 2.905	3.216 to 3.249
HillSlope	8.359 to 11.32		16.55 to 18.63	67.05 to 89.16	3.803 to 7.173	9.150 to 26.64
EC50	51.13 to 52.19		178.3 to 179.9	313.1 to 314.5	708.1 to 802.9	1644 to 1776
Goodness of Fit						
Degrees of Freedom	4		9	13	17	26
R square	0.9997		0.9997	0.9987	0.9199	0.9322
Absolute Sum of Squares	3.48		5.88	25	197.2	1642
Sy.x	0.9327		0.8083	1.387	3.406	7.947
Runs test						
Points above curve	3		3	2	1	7
Points below curve	3		8	13	18	21
Number of runs	4		4	5	3	4
P value (runs test)	0.7		0.2364	1	1	0.0002
Deviation from Model	Not Significant		Not Significant	Not Significant	Not Significant	Significant
Number of points						
Analyzed	6	7	11	15	19	28
	LogEC50		HillSlope		EC50	
	Value	SE	Value	SE	Value	SE
L1	1.713144	0.001607007	9.841871	0.5342584	51.65881	
L2	2.138497		122.8396		137.5615	
L3f	2.253009	0.000848848	17.59011	0.4598216	179.0644	
L3m	2.496633	0.00046653	78.10342	5.118027	313.7855	
P	2.877389	0.0129326	5.487879	0.7987151	754.0303	
A	3.232617	0.008174426	17.89533	4.253516	1708.507	

**Dataset**						
* starred and italicized data points were excluded from final regression						
log(time)	L1	L2	L3f	L3m	P	A
1.236054	0	0	0	0	0	0
1.533867	0	0	0	0	0	0
1.709482	48.2	0	0	0	0	0
1.833864	93.2	0	0	0	0	0
1.98802	100	0	0	0	0	0
2.053974	100	0	0	0	0	0
2.149553		95.8	0	0	0	0
2.228101			27.1	0	0	0
2.294751			83.8	0	0	0
2.352303			100	0	0	0
2.421796			100	0	0	0
2.481622				6.3	0	0
2.502478				74.1	0	0
2.581053				100	0	0
2.647473				95	5	0
2.691148					3	0
2.751722					16.3	0
2.801432					38.5	0
2.847614					34.3	0
2.889302					*5.0**	0
2.974162					*15.1**	28.6
3.044899					*34.9**	26.6
3.106384					*56.2**	11.2
3.159266					*64.1**	5.3
3.206861						24.6
3.272534						84.4
3.329401						100
3.379864						100

Appendix C4. *L. sericata* 17.5° C.

***L. sericata* 17.5° C**	L1	L2	L3f	L3m	P	A
log(agonist) vs. normalized response -- Variable slope						
Best-fit values						
LogEC50	1.659	1.932	2.135	2.375	2.514	2.881
HillSlope	3.5	30.37	28.04	18.39	13.65	15.91
EC50	45.63	85.43	136.6	237.2	326.4	760.8
Std. Error						
LogEC50	0.03999	0.00945	0.001194	0.0004714	0.006797	0.001571
HillSlope	1.169	8.3	4.781	0.3376	2.473	0.8792
95% Confidence Intervals						
LogEC50	1.532 to 1.786	1.908 to 1.955	2.133 to 2.138	2.374 to 2.376	2.499 to 2.528	2.878 to 2.884
HillSlope	-0.2189 to 7.219	10.06 to 50.68	17.22 to 38.85	17.66 to 19.12	8.433 to 18.87	14.09 to 17.72
EC50	34.04 to 61.16	81.00 to 90.10	135.7 to 137.4	236.7 to 237.8	315.8 to 337.3	755.1 to 766.5
Goodness of Fit						
Degrees of Freedom	3	6	9	13	17	24
R square	0.9333	0.9972	0.998	0.9997	0.9691	0.9981
Absolute Sum of Squares	386.3	38.36	32.9	4.36	660.4	60.32
Sy.x	11.35	2.528	1.912	0.5791	6.233	1.585
Runs test						
Points above curve	2	1	3	2	1	3
Points below curve	3	7	8	13	18	23
Number of runs	4	3	4	4	3	6
P value (runs test)	0.9	1	0.2364	0.3714	1	0.4077
Deviation from Model	Not Significant	Not Significant	Not Significant	Not Significant	Not Significant	Not Significant
Number of points						
Analyzed	5	8	11	15	19	26
	LogEC50		HillSlope		EC50	
	Value	SE	Value	SE	Value	SE
L1	1.65922	0.03998688	3.499854	1.168679	45.62676	
L2	1.931607	0.009449857	30.36921	8.300381	85.42932	
L3f	2.135411	0.001194284	28.03586	4.781146	136.5873	
L3m	2.375128	0.000471389	18.39026	0.3376454	237.2071	
P	2.513714	0.006797008	13.65121	2.473212	326.373	
A	2.881255	0.001571311	15.90591	0.879162	760.7725	

**Dataset**						
* starred and italicized data points were excluded from final regression						
log(time)	L1	L2	L3f	L3m	P	A
1.081887	0	0	0	0	0	0
1.383217	0	0	0	0	0	0
1.558859	44.2	0	0	0	0	0
1.684022	45.2	0	0	0	0	0
1.779476		0	0	0	0	0
1.898176	91.2	8.8	0	0	0	0
1.99189		98.6	0	0	0	0
2.068371		93.8	6.3	0	0	0
2.133832			46.9	0	0	0
2.190588			100	0	0	0
2.257878			100	0	0	0
2.316198				6.3	0	0
2.36748				42.9	0	0
2.413481				82.5	0	0
2.4552				97.2	2.8	0
2.522575					69.6	0
2.581034					73.6	0
2.632525					93.8	0
2.67859					89.3	0
2.720221						0
2.802805						0
2.872212						44.3
2.931958						83.3
2.98455						100
3.031408						96.4
3.103176						100

Appendix C5. *L. sericata* 20.0° C.

***L. sericata* 20.0° C**	L1	L2	L3f	L3m	P	A
log(agonist) vs. normalized response -- Variable slope	Interrupted	Interrupted			Interrupted	Ambiguous
Best-fit values						
LogEC50	1.447	1.763	1.964	2.277	2.336	~ 2.743
HillSlope	33.46	98.41	36.98	75.89	215.3	~ 127.2
EC50	28.01	57.92	92.11	189.3	217	~ 553.4
Std. Error						
LogEC50			0.00001851	0.0207		~ 259.2
HillSlope			0.0264	120.9		~ 4.395e+006
95% Confidence Intervals						
LogEC50			1.964 to 1.964	2.232 to 2.322		(Very wide)
HillSlope			36.92 to 37.04	-185.3 to 337.1		(Very wide)
EC50			92.10 to 92.12	170.8 to 209.8		(Very wide)
Goodness of Fit						
Degrees of Freedom			9	13		23
R square			1	0.9886		0.9996
Absolute Sum of Squares			0.00115	156.2		13.69
Sy.x			0.01131	3.466		0.7715
Runs test						
Points above curve			3	1		2
Points below curve			8	14		22
Number of runs			4	3		3
P value (runs test)			0.2364	1		0.087
Deviation from Model			Not Significant	Not Significant		Not Significant
Number of points						
Analyzed	3	7	11	15	17	25
	LogEC50		HillSlope		EC50	
	Value	SE	Value	SE	Value	SE
L1	1.447243		33.45631		28.00549	
L2	1.762846		98.41061		57.92239	
L3f	1.964321	1.85109E-05	36.97882	0.02640347	92.11302	
L3m	2.277081	0.02070029	75.88952	120.9227	189.2694	
P	2.336397		215.3207		216.9686	
A	2.743006	259.2079	127.1961	4394835	553.3578	

**Dataset**						
* starred and italicized data points were excluded from final regression						
log(time)	L1	L2	L3f	L3m	P	A
0.956649	0	0	0	0	0	0
1.257278	0	0	0	0	0	0
1.435367	28.6	0	0	0	0	0
1.557607		0	0	0	0	0
1.655018		0	0	0	0	0
1.771434		87.5	0	0	0	0
1.864783		100	0	0	0	0
1.940122			11.3	0	0	0
2.004751			96.9	0	0	0
2.060934			100	0	0	0
2.130602			100	0	0	0
2.190495				0	0	0
2.243172				0	0	0
2.290035				90.6	0	0
2.332472				87.5	12.5	0
2.399703					100	0
2.457882					100	0
2.509225						0
2.555094						0
2.596597						0
2.680336						0
2.750508						90
2.810904						100
2.863917						96.3
2.911158						100

Appendix C6. *L. sericata* 22.5 C.

L. sericata **22.5° C**	L1	L2	L3f	L3m	P	A
log(agonist) vs. normalized response -- Variable slope		Interrupted				
Best-fit values						
LogEC50	1.345	1.66	1.889	2.15	2.306	2.628
HillSlope	13.31	104.8	30.03	12.03	26.05	77.39
EC50	22.13	45.74	77.51	141.1	202.4	424.6
Std. Error						
LogEC50	0.009957		0.001529	0.006895	0.0002226	0.2165
HillSlope	5.194		4.503	2.114	0.4109	884
95% Confidence Intervals						
LogEC50	1.313 to 1.377		1.886 to 1.893	2.135 to 2.165	2.306 to 2.307	2.180 to 3.076
HillSlope	-3.220 to 29.84		20.00 to 40.07	7.465 to 16.60	25.18 to 26.91	-1752 to 1906
EC50	20.57 to 23.81		76.91 to 78.12	136.4 to 146.1	202.2 to 202.6	151.3 to 1191
Goodness of Fit						
Degrees of Freedom	3		10	13	17	23
R square	0.9897		0.9991	0.9532	0.9999	0.9985
Absolute Sum of Squares	89.51		21.02	573	1.302	48.89
Sy.x	5.462		1.45	6.639	0.2768	1.458
Runs test						
Points above curve	2		3	1	3	2
Points below curve	3		9	14	16	23
Number of runs	5		4	3	4	4
P value (runs test)	1		0.2	1	0.0815	0.23
Deviation from Model	Not Significant		Not Significant	Not Significant	Not Significant	Not Significant
Number of points						
Analyzed	5	8	12	15	19	25
	LogEC50		HillSlope		EC50	
	Value	SE	Value	SE	Value	SE
L1	1.34501	0.009957033	13.30862	5.194256	22.13144	
L2	1.6603		104.7518		45.7404	
L3f	1.889368	0.001528542	30.03393	4.502685	77.51189	
L3m	2.149634	0.006894524	12.03136	2.113842	141.1347	
P	2.306204	0.000222566	26.04704	0.4108769	202.397	
A	2.62794	0.2165114	77.39233	884.0189	424.5608	

Dataset

* starred and italicized data points were excluded from final regression

log(time)	L1	L2	L3f	L3m	P	A
0.848446	0	0	0	0	0	0
1.151906	5	0	0	0	0	0
1.325652	35.4	0	0	0	0	0
1.450313	97.5	0	0	0	0	0
1.545256	91.7	0	0	0	0	0
1.664093		71.4	0	0	0	0
1.756953		100	0	0	0	0
1.832961		100	0	0	0	0
1.897902			64.7	0	0	0
1.954665			94.8	0	0	0
2.025562			100	0	0	0
2.086745			100	0	0	0
2.140167				59.4	0	0
2.188026				66.7	0	0
2.230609				85	0	0
2.296894					36.5	0
2.354229					94.4	0
2.404834					100	0
2.450275					100	0
2.491385						0
2.576341						0
2.646894						96.7
2.708421						98
2.761928						93.3
2.813581						100

Appendix C7. *L. sericata* 25.0° C.

***L. sericata* 25.0° C**	L1	L2	L3f	L3m	P	A
log(agonist) vs. normalized response -- Variable slope		Interrupted				
Best-fit values						
LogEC50	1.228	1.585	1.794	2.047	2.225	2.568
HillSlope	11.19	102	20.2	60.17	20.13	25.89
EC50	16.92	38.44	62.3	111.3	167.8	370.2
Std. Error						
LogEC50	0.0144		0.0007994	0.003992	0.001102	0.001036
HillSlope	4.173		0.5171	22.69	1.094	3.598
95% Confidence Intervals						
LogEC50	1.183 to 1.274		1.793 to 1.796	2.038 to 2.055	2.222 to 2.227	2.566 to 2.571
HillSlope	-2.088 to 24.47		19.03 to 21.37	10.72 to 109.6	17.82 to 22.44	18.45 to 33.34
EC50	15.23 to 18.81		62.04 to 62.56	109.1 to 113.6	166.9 to 168.7	368.3 to 372.0
Goodness of Fit						
Degrees of Freedom	3		9	12	17	23
R square	0.9937		0.9997	0.9994	0.9988	0.9981
Absolute Sum of Squares	58.91		5.836	7.735	27.88	49.69
Sy.x	4.431		0.8053	0.8028	1.281	1.47
Runs test						
Points above curve	1		3	1	2	2
Points below curve	4		8	13	17	23
Number of runs	3		4	3	4	4
P value (runs test)	1		0.2364	1	0.2982	0.23
Deviation from Model	Not Significant		Not Significant	Not Significant	Not Significant	Not Significant
Number of points						
Analyzed	5	7	11	14	19	25
	LogEC50		HillSlope		EC50	
	Value	SE	Value	SE	Value	SE
L1	1.228471	0.01440128	11.18917	4.172692	16.92274	
L2	1.584817		102.0207		38.44302	
L3f	1.794457	0.00079943	20.20193	0.5170847	62.29551	
L3m	2.046651	0.003991658	60.16519	22.68968	111.3398	
P	2.224674	0.001101964	20.12869	1.09356	167.7542	
A	2.568397	0.001036366	25.8913	3.598356	370.1664	

**Dataset**						
* starred and italicized data points were excluded from final regression						
log(time)	L1	L2	L3f	L3m	P	A
0.785626	0	0	0	0	0	0
1.083682	0	0	0	0	0	0
1.26156	70.7	0	0	0	0	0
1.383666	94.8	0	0	0	0	0
1.479167	93.4	0	0	0	0	0
1.593101		87.5	0	0	0	0
1.683947		100	0	0	0	0
1.756826			15.2	0	0	0
1.820119			76.2	0	0	0
1.875592			100	0	0	0
1.944996			100	0	0	0
2.004518				0	0	0
2.057206				81.2	0	0
2.104259				97.2	0	0
2.146967					0	0
2.215571					40.1	0
2.274341					90.8	0
2.326583					94.6	0
2.372912					100	0
2.414973						0
2.499687						0
2.570543						53.4
2.631461						96.9
2.684845						93.1
2.732394						100

Appendix C8. *L. sericata* 27.5° C.

***L. sericata* 27.5° C**	L1	L2	L3f	L3m	P	A
log(agonist) vs. normalized response -- Variable slope						
Best-fit values						
LogEC50	1.073	1.489	1.667	2.094	2.197	2.537
HillSlope	2.373	29.23	74.36	22.77	23.07	14.99
EC50	11.83	30.87	46.5	124.3	157.6	344.5
Std. Error						
LogEC50	0.05188	0.0267	0.4354	0.001935	0.00195	0.001775
HillSlope	0.6716	24.71	1324	2.179	2.435	0.7553
95% Confidence Intervals						
LogEC50	0.9080 to 1.238	1.421 to 1.558	0.6635 to 2.671	2.090 to 2.099	2.193 to 2.202	2.533 to 2.541
HillSlope	0.2357 to 4.510	-34.31 to 92.77	-2980 to 3128	18.10 to 27.44	17.93 to 28.21	13.43 to 16.56
EC50	8.092 to 17.31	26.35 to 36.15	4.608 to 469.3	123.1 to 125.5	156.1 to 159.1	341.6 to 347.4
Goodness of Fit						
Degrees of Freedom	3	5	8	14	17	23
R square	0.9174	0.9988	0.9963	0.9932	0.9947	0.9969
Absolute Sum of Squares	359.6	15.11	71.45	58.13	97.76	66.53
Sy.x	10.95	1.738	2.989	2.038	2.398	1.701
Runs test						
Points above curve	2	1	1	1	2	3
Points below curve	3	6	9	15	17	22
Number of runs	3	3	3	3	4	4
P value (runs test)	0.5	1	1	1	0.2982	0.0474
Deviation from Model	Not Significant	Not Significant	Not Significant	Not Significant	Not Significant	Significant
Number of points						
Analyzed	5	7	10	16	19	25
	LogEC50		HillSlope		EC50	
	Value	SE	Value	SE	Value	SE
L1	1.073115	0.05187581	2.372677	0.6715761	11.83355	
L2	1.489477	0.0266954	29.23228	24.71381	30.86573	
L3f	1.667488	0.4353856	74.35593	1324.423	46.50373	
L3m	2.094481	0.001934841	22.77118	2.178688	124.3028	
P	2.197497	0.001950332	23.07309	2.435186	157.5786	
A	2.537146	0.001775475	14.99456	0.7553464	344.4659	

**Dataset**						
* starred and italicized data points were excluded from final regression						
log(time)	L1	L2	L3f	L3m	P	A
0.704722	0	0	0	0	0	0
1.005931	48.9	0	0	0	0	0
1.18327	70.1	0	0	0	0	0
1.306514	66.9	0	0	0	0	0
1.400754	85.4	0	0	0	0	0
1.520975		89.3	0	0	0	0
1.615117		96.1	0	0	0	0
1.691928			98.5	0	0	0
1.756699			91.7	0	0	0
1.813581			98.4	0	0	0
1.881504				0	0	0
1.93981				0	0	0
1.991595				0	0	0
2.041787				0	0	0
2.082785				37.5	0	0
2.149591				90.6	3.1	0
2.207051					65	0
2.257679					87.5	0
2.303196					100	0
2.344392						0
2.42984						0
2.501059						25
2.562293						66.7
2.61595						100
2.663701						100

Appendix C9. *L. sericata* 30.0° C.

***L. sericata* 30.0° C**	L1	L2	L3f	L3m	P	A
log(agonist) vs. normalized response -- Variable slope		Ambiguous				
Best-fit values						
LogEC50	1.058	~ 1.391	1.619	1.938	2.144	2.473
HillSlope	3.885	~ 360.1	14.4	67.55	16.63	32.25
EC50	11.44	~ 24.60	41.56	86.67	139.3	297.2
Std. Error						
LogEC50	0.04688	~	0.002951	0.006475	0.001078	0.004462
HillSlope	1.806	~	1.466	55.44	0.644	4.831
95% Confidence Intervals						
LogEC50	0.8566 to 1.260	(Very wide)	1.612 to 1.625	1.924 to 1.952	2.142 to 2.146	2.464 to 2.482
HillSlope	-3.887 to 11.66	(Very wide)	11.09 to 17.72	-52.21 to 187.3	15.28 to 17.98	22.25 to 42.25
EC50	7.188 to 18.20	(Very wide)	40.93 to 42.21	83.93 to 89.51	138.5 to 140.0	290.9 to 303.6
Goodness of Fit						
Degrees of Freedom	2	5	9	13	19	23
R square	0.8853	1	0.9967	0.9989	0.9992	0.985
Absolute Sum of Squares	424.3	0	63.53	17.63	25.17	333.5
Sy.x	14.57	5.942E-30	2.657	1.165	1.151	3.808
Runs test						
Points above curve	1	1	3	2	3	2
Points below curve	3	5	8	13	18	23
Number of runs	3	2	4	5	6	4
P value (runs test)	1	0.3333	0.2364	1	0.4887	0.23
Deviation from Model	Not Significant	Not Significant	Not Significant	Not Significant	Not Significant	Not Significant
Number of points						
Analyzed	4	7	11	15	21	25
	LogEC50		HillSlope		EC50	
	Value	SE	Value	SE	Value	SE
L1	1.058351	0.04687951	3.884845	1.806252	11.43803	
L2	1.390995		360.1489		24.60338	
L3f	1.61869	0.002950681	14.40494	1.466204	41.56142	
L3m	1.937892	0.006474931	67.54924	55.44376	86.67464	
P	2.143842	0.00107768	16.62763	0.6440066	139.2652	
A	2.473039	0.00446208	32.24966	4.831073	297.1935	

**Dataset**						
* starred and italicized data points were excluded from final regression						
log(time)	L1	L2	L3f	L3m	P	A
0.613225	0	0	0	0	0	0
0.909601	13.5	0	0	0	0	0
1.084427	70.3	0	0	0	0	0
1.183507	62.9	0	0	0	0	0
1.305262		0	0	0	0	0
1.43397		100	0	0	0	0
1.534079		100	0	0	0	0
1.615775			50.4	0	0	0
1.68331			85	0	0	0
1.741644			100	0	0	0
1.813803			100	0	0	0
1.87629				2.6	0	0
1.930015				22.7	0	0
1.978181				100	0	0
2.004608				96.7	0	0
2.089905					13.5	0
2.149412					53.3	0
2.202159					93.8	0
2.247973					96.8	0
2.290035					98.3	0
2.374809					100	0
2.445604						11.3
2.507856						93.8
2.559907						81.6
2.607455						100

Appendix C10. *L. sericata* 32.5° C.

***L. sericata* 32.5° C**	L1	L2	L3f	L3m	P	A
log(agonist) vs. normalized response -- Variable slope		Interrupted				
Best-fit values						
LogEC50	1.139	1.4	1.619	1.978	2.252	2.543
HillSlope	4.308	110.3	14.19	10.99	6.571	11.48
EC50	13.77	25.11	41.59	95.09	178.6	349.1
Std. Error						
LogEC50	0.009731		0.003305	0.01157	0.008096	0.007449
HillSlope	0.4771		1.079	3.094	0.7149	2.115
95% Confidence Intervals						
LogEC50	1.108 to 1.170		1.611 to 1.627	1.953 to 2.003	2.235 to 2.269	2.528 to 2.558
HillSlope	2.789 to 5.826		11.71 to 16.68	4.350 to 17.62	5.084 to 8.058	7.119 to 15.85
EC50	12.82 to 14.79		40.86 to 42.32	89.81 to 100.7	171.8 to 185.6	337.0 to 361.7
Goodness of Fit						
Degrees of Freedom	3		8	14	21	24
R square	0.9917		0.9996	0.8745	0.9719	0.9137
Absolute Sum of Squares	39.59		6.807	1458	575.4	996.9
Sy.x	3.633		0.9224	10.2	5.235	6.445
Runs test						
Points above curve	2		2	2	3	2
Points below curve	3		8	14	20	24
Number of runs	4		4	4	4	4
P value (runs test)	0.9		0.5333	0.35	0.0559	0.2215
Deviation from Model	Not Significant		Not Significant	Not Significant	Not Significant	Not Significant
Number of points						
Analyzed	5	7	10	16	23	26
	LogEC50		HillSlope		EC50	
	Value	SE	Value	SE	Value	SE
L1	1.139	0.009731171	4.307749	0.4771413	13.77211	
L2	1.399831		110.2617		25.10909	
L3f	1.618942	0.003305214	14.19396	1.079273	41.58552	
L3m	1.978156	0.01156718	10.98604	3.093692	95.09459	
P	2.251843	0.008095888	6.571046	0.7148767	178.5843	
A	2.542994	0.007449316	11.48417	2.114877	349.1358	

**Dataset**						
* starred and italicized data points were excluded from final regression						
log(time)	L1	L2	L3f	L3m	P	A
0.611458	0	0	0	0	0	0
0.910491	5.8	0	0	0	0	0
1.094442	43.3	0	0	0	0	0
1.182795	57.7	0	0	0	0	0
1.285932	81.3	0	0	0	0	0
1.402046		63.7	0	0	0	0
1.494039		100	0	0	0	0
1.592732			*90.0**	0	0	0
1.655178			77.2	0	0	0
1.708633			93.1	0	0	0
1.779356			100	0	0	0
1.840603				0	0	0
1.892813				0	0	0
1.940641				52.9	0	0
1.983043				30.3	0	0
2.049735				100	0	0
2.092165					0	0
2.158928					11.1	0
2.20438					36.2	0
2.245831					63.1	0
2.328677					66	0
2.398099					82.8	0
2.439484					96.7	0
2.495035						41.7
2.542825						30.9
2.61066						100

APPENDIX D

Datasets and statistical analysis of 1/day versus temperature for determining degree day coefficients. Iterative testing for non-linearity (a violation of degree-day assumptions) resulted in some data points being excluded, as indicated. The analysis is from GraphPad Prism 6.0.

***L. sericata* 1/days vs. temperature**												
Dataset												
* data points with an asterick and italics were excluded from final regression (because they were on the non-linear part of development curve or were not biologically meaningful)												
Temp	E-L1	E-L2	E-L3f	E-L3m	E-P	E-A	Egg	L1	L2	L3f	L3m	P
10.4	*0.155**	*0.076**	*0.053**	*0.035**	*0.017**	*0.006**	*0.155**	*0.148**	*0.179**	*0.103**	*0.034**	*0.010**
12.7	*0.742**	0.176	0.104	0.056	0.012	0.008	*0.742**	0.231	0.254	0.12	0.016	0.026
15.1	0.465	0.174	0.134	0.076	0.032	0.014	0.465	0.279	0.578	0.178	0.055	0.025
17.5	0.526	0.281	0.176	0.101	0.074	0.032	0.526	0.603	0.469	0.239	0.269	0.055
20.1	0.857	0.414	0.261	0.127	0.111	0.043	0.857	0.802	0.702	0.247	*0.866**	0.071
22.5	1.084	0.525	0.31	0.17	0.119	0.057	1.084	1.017	0.755	0.377	0.392	0.108
25	1.418	0.624	0.385	0.216	0.143	0.065	1.418	1.115	1.006	0.489	0.425	0.119
27.5	2.028	0.778	0.516	0.193	0.152	0.07	2.028	1.261	1.535	0.308	0.721	0.128
30	2.098	0.975	0.577	0.277	0.172	0.081	2.098	1.823	1.415	0.532	0.456	0.152
32.5	*1.743**	*0.956**	*0.664**	*0.252**	*0.134**	*0.069**	*1.743**	*2.117**	*2.174**	*0.407**	*0.287**	*0.141**
	E-L1	E-L2	E-L3f	E-L3m	E-P	E-A	Egg	L1	L2	L3f	L3m	P
Best-fit values												
Slope	0.1217 ± 0.01092	0.04694 ± 0.003395	0.02838 ± 0.001918	0.01212 ± 0.001174	0.009298 ± 0.0007915	0.004344 ± 0.0002483	0.1217 ± 0.01092	0.08563 ± 0.007374	0.07014 ± 0.009861	0.02120 ± 0.004594	0.03353 ± 0.007493	0.007817 ± 0.0005470
Y-intercept when X=0.0	-1.530 ± 0.2520	-0.5064 ± 0.07483	-0.2966 ± 0.04229	-0.1061 ± 0.02588	-0.09618 ± 0.01745	-0.04628 ± 0.005472	-1.530 ± 0.2520	-0.9326 ± 0.1625	-0.6547 ± 0.2174	-0.1403 ± 0.1013	-0.3864 ± 0.1672	-0.08099 ± 0.01206
X-intercept when Y=0.0	12.58	10.79	10.45	8.756	10.34	10.65	12.58	10.89	9.334	6.617	11.53	10.36
1/slope	8.219	21.31	35.24	82.53	107.5	230.2	8.219	11.68	14.26	47.17	29.83	127.9
95% Confidence Intervals												
Slope	0.09358 to 0.1497	0.03863 to 0.05524	0.02368 to 0.03307	0.009245 to 0.01499	0.007362 to 0.01124	0.003737 to 0.004952	0.09358 to 0.1497	0.06759 to 0.1037	0.04601 to 0.09427	0.009958 to 0.03244	0.01426 to 0.05279	0.006478 to 0.009155
Y-intercept when X=0.0	-2.178 to -0.8822	-0.6895 to -0.3232	-0.4000 to -0.1931	-0.1694 to -0.04278	-0.1389 to -0.05349	-0.05967 to -0.03289	-2.178 to -0.8822	-1.330 to -0.5349	-1.187 to -0.1228	-0.3881 to 0.1075	-0.8162 to 0.04330	-0.1105 to -0.05149
X-intercept when Y=0.0	9.318 to 14.71	8.273 to 12.62	8.069 to 12.22	4.568 to 11.45	7.173 to 12.52	8.724 to 12.16	9.318 to 14.71	7.806 to 13.01	2.616 to 12.84	-10.52 to 12.27	-2.906 to 16.15	7.865 to 12.20
Goodness of Fit												
R square	0.9613	0.9696	0.9733	0.9467	0.9583	0.9808	0.9613	0.9574	0.894	0.7802	0.8001	0.9715
Sy.x	0.1437	0.05448	0.03079	0.01884	0.0127	0.003984	0.1437	0.1183	0.1583	0.07372	0.1199	0.008778
Is slope significantly non-zero?												
F	124.1	191.1	218.8	106.5	138	306.2	124.1	134.9	50.59	21.3	20.02	204.2
DFn, DFd	1.000, 5.000	1.000, 6.000	1.000, 6.000	1.000, 6.000	1.000, 6.000	1.000, 6.000	1.000, 5.000	1.000, 6.000	1.000, 6.000	1.000, 6.000	1.000, 5.000	1.000, 6.000
P value	0.0001	< 0.0001	< 0.0001	< 0.0001	< 0.0001	< 0.0001	0.0001	< 0.0001	0.0004	0.0036	0.0066	< 0.0001
Deviation from zero?	Significant	Significant	Significant	Significant	Significant	Significant	Significant	Significant	Significant	Significant	Significant	Significant
Data												
Number of X values	7	8	8	8	8	8	7	8	8	8	7	8
Maximum number of Y replicates	1	1	1	1	1	1	1	1	1	1	1	1
Total number of values	7	8	8	8	8	8	7	8	8	8	7	8
Number of missing values	3	2	2	2	2	2	3	2	2	2	3	2
Runs test												
Points above line	2	2	4	4	4	4	2	5	3	4	3	3
Points below line	5	6	4	4	4	4	5	3	5	4	4	5
Number of runs	4	3	3	5	3	3	4	5	4	6	5	4
P value (runs test)	0.7143	0.2857	0.1143	0.6286	0.1143	0.1143	0.7143	0.7143	0.4286	0.8857	0.8	0.4286
Deviation from linearity	Not Significant	Not Significant	Not Significant	Not Significant	Not Significant	Not Significant	Not Significant	Not Significant	Not Significant	Not Significant	Not Significant	Not Significant
Equation	Y = 0.1217*X - 1.530	Y = 0.04694*X - 0.5064	Y = 0.02838*X - 0.2966	Y = 0.01212*X - 0.1061	Y = 0.009298*X - 0.09618	Y = 0.004344*X - 0.04628	Y = 0.1217*X - 1.530	Y = 0.08563*X - 0.9326	Y = 0.07014*X - 0.6547	Y = 0.02120*X - 0.1403	Y = 0.03353*X - 0.3864	Y = 0.007817*X - 0.08099

APPENDIX E

Datasets and statistical analysis of degree days versus temperature. For degree days to be valid the linear regression should have zero slope. The analysis is from GraphPad Prism 6.0.

L. sericata degree day analysis

* data points with an asterix and italics were excluded from final regression (because they were on the non-linear part of development curve or were not biologically meaningful)

	ADD versus temperature						DD versus temperature					
Temperature	E-L1	E-L2	E-L3f	E-L3m	E-P	E-A	Egg	L1	L2	L3f	L3m	P
15.1	5.33	*24.45**	34.36	82.36			5.3	14.9	9.9	47.4		
17.5	9.42	24	40.31	86.74	97.81	218.17	9.4	11	17.5	45.8	22.3	129.8
20.1	8.75	22.41	36.95	89.28	88.02	217.35	8.7	11.5	15.3	54.5		136.2
22.5	9.18	22.39	39.05	81.06	102.89	210.33	9.2	11.5	17.5	42.2	28.1	112.7
25	8.75	22.75	37.74	75.31	102.4	221.17	8.8	12.6	15.6	37.5	31.6	123.4
27.5	7.36	21.51	33.07	97.17	*112.78**	*242.08**	7.4	13.2	11.8	67.7	22.2	133.6
30	8.31	*19.70**	33.87	*76.76**	*114.14**	*239.74**	8.3	10.5	14.6	44	*40.5**	129.3
	E-L1	E-L2	E-L3f	E-L3m	E-P	E-A	Egg	L1	L2	L3f	L3m	P
Best-fit values												
Slope	0.06836 ± 0.1137	-0.1874 ± 0.07593	-0.2200 ± 0.2086	0.3664 ± 0.7921	1.123 ± 1.293	0.08174 ± 1.007	0.07236 ± 0.1137	-0.1101 ± 0.1161	0.04104 ± 0.2354	0.2428 ± 0.8215	0.2446 ± 0.7445	0.01261 ± 0.9067
Y-intercept when X=0.0	6.617 ± 2.624	26.83 ± 1.731	41.44 ± 4.813	77.52 ± 17.19	73.89 ± 27.73	215.0 ± 21.61	6.527 ± 2.624	14.65 ± 2.678	13.68 ± 5.432	42.97 ± 18.95	20.39 ± 17.43	127.2 ± 21.89
X-intercept when Y=0.0	-96.8	143.2	188.3	-211.6	-65.79	-2631	-90.21	133.1	-333.2	-177	-83.39	-10086
1/slope	14.63	-5.336	-4.545	2.73	0.8904	12.23	13.82	-9.085	24.37	4.119	4.089	79.29
95% Confidence Intervals												
Slope	-0.2240 to 0.3607	-0.4290 to 0.05421	-0.7563 to 0.3163	-1.832 to 2.565	-4.439 to 6.685	-4.253 to 4.416	-0.2201 to 0.3648	-0.4085 to 0.1884	-0.5643 to 0.6463	-1.869 to 2.355	-2.959 to 3.448	-2.504 to 2.530
Y-intercept when X=0.0	-0.1280 to 13.36	21.33 to 32.34	29.06 to 53.81	29.81 to 125.2	-45.45 to 193.2	122.0 to 308.0	-0.2194 to 13.27	7.766 to 21.54	-0.2889 to 27.64	-5.755 to 91.70	-54.63 to 95.41	66.43 to 188.0
X-intercept when Y=0.0	-infinity to 0.3619	75.16 to +infinity	70.58 to +infinity	-infinity to -11.75	-infinity to 6.870	-infinity to -27.73	-infinity to 0.6133	52.02 to +infinity	-infinity to 0.4573	-infinity to 2.503	-infinity to 16.46	-infinity to -26.46
Goodness of Fit												
R square	0.06739	0.67	0.182	0.05077	0.274	0.003282	0.07488	0.1524	0.00604	0.01716	0.0512	0.00004836
Sy.x	1.496	0.5979	2.744	8.227	7.197	5.609	1.496	1.527	3.097	10.81	5.505	9.451
Is slope significantly non-zero?												
F	0.3613	6.091	1.112	0.2139	0.755	0.006585	0.4047	0.8991	0.03038	0.08732	0.1079	0.0001935
DFn, DFd	1.000, 5.000	1.000, 3.000	1.000, 5.000	1.000, 4.000	1.000, 2.000	1.000, 2.000	1.000, 5.000	1.000, 5.000	1.000, 5.000	1.000, 5.000	1.000, 2.000	1.000, 4.000
P value	0.574	0.0902	0.3398	0.6677	0.4765	0.9427	0.5527	0.3866	0.8685	0.7795	0.7737	0.9896
Deviation from zero?	Not Significant	Not Significant	Not Significant	Not Significant	Not Significant	Not Significant	Not Significant	Not Significant	Not Significant	Not Significant	Not Significant	Not Significant
Data												
Number of X values	7	5	7	6	4	4	7	7	7	7	4	6
Maximum number of Y replicates	1	1	1	1	1	1	1	1	1	1	1	1
Total number of values	7	5	7	6	4	4	7	7	7	7	4	6
Number of missing values	0	2	0	1	3	3	0	0	0	0	3	1
Runs test												
Points above line	4	2	3	3	3	3	4	3	4	3	2	4
Points below line	3	3	4	3	1	1	3	4	3	4	2	2
Number of runs	3	4	5	4	3	3	3	4	3	6	3	3
P value (runs test)	0.2	0.9	0.8	0.7	1	1	0.2	0.5429	0.2	0.9714	0.6667	0.4
Deviation from linearity	Not Significant	Not Significant	Not Significant	Not Significant	Not Significant	Not Significant	Not Significant	Not Significant	Not Significant	Not Significant	Not Significant	Not Significant
